# A comprehensive analysis of *Helicobacter pylori* plasticity zones reveals that they are integrating conjugative elements with intermediate integration specificity

**DOI:** 10.1186/1471-2164-15-310

**Published:** 2014-04-27

**Authors:** Wolfgang Fischer, Ute Breithaupt, Beate Kern, Stella I Smith, Carolin Spicher, Rainer Haas

**Affiliations:** 1 Max von Pettenkofer-Institut für Hygiene und Medizinische Mikrobiologie, Ludwig-Maximilians-Universität, D-80336 Munich, Germany; 2 Molecular Biology and Biotechnology Division, Nigerian Institute of Medical Research, Yaba, PMB2013 Lagos, Nigeria

**Keywords:** Plasticity zone, *Helicobacter pylori*, Integrating conjugative element, Type IV secretion system, Horizontal gene transfer

## Abstract

**Background:**

The human gastric pathogen *Helicobacter pylori* is a paradigm for chronic bacterial infections. Its persistence in the stomach mucosa is facilitated by several mechanisms of immune evasion and immune modulation, but also by an unusual genetic variability which might account for the capability to adapt to changing environmental conditions during long-term colonization. This variability is reflected by the fact that almost each infected individual is colonized by a genetically unique strain. Strain-specific genes are dispersed throughout the genome, but clusters of genes organized as genomic islands may also collectively be present or absent.

**Results:**

We have comparatively analysed such clusters, which are commonly termed plasticity zones, in a high number of *H. pylori* strains of varying geographical origin. We show that these regions contain fixed gene sets, rather than being true regions of genome plasticity, but two different types and several subtypes with partly diverging gene content can be distinguished. Their genetic diversity is incongruent with variations in the rest of the genome, suggesting that they are subject to horizontal gene transfer within *H. pylori* populations. We identified 40 distinct integration sites in 45 genome sequences, with a conserved heptanucleotide motif that seems to be the minimal requirement for integration.

**Conclusions:**

The significant number of possible integration sites, together with the requirement for a short conserved integration motif and the high level of gene conservation, indicates that these elements are best described as integrating conjugative elements (ICEs) with an intermediate integration site specificity.

## Background

Infections with the human gastric pathogen *H. pylori* are paradigmatic examples of chronic, or persistent, bacterial infections in the face of a constant immune response [1]. *H. pylori* infections are usually contracted during early childhood and persist for the lifetime of the host, but most infected individuals develop only mild gastric inflammation without overt symptoms. Nevertheless, a substantial fraction of infected persons develops more severe consequences, making *H. pylori* the principal cause of (symptomatic) chronic active gastritis and peptic ulcer disease, and a major risk factor for development of gastric adenocarcinoma and mucosa-associated lymphoid tissue (MALT) lymphoma [2,3]. For survival and persistent growth in the presence of a constant immune response and in an environment which is changing considerably over decades of infection, permanent adaptation of the bacteria is thought to be required [4]. Such adaptive processes may include regulatory mechanisms acting on gene expression, but also reversible or irreversible genome changes. For instance, it has been shown that strains isolated from patients with atrophic gastritis [5] or marginal zone B-cell MALT lymphoma [6] have reduced genomes in comparison to gastritis or ulcer strains, and a strain isolated from a gastric cancer patient had lost further genes in comparison to a strain isolated previously from the same patient during atrophic gastritis [7]. That genome plasticity plays a role in bacterial persistence is further supported by the observation that natural transformation competence, which is upregulated upon DNA stress [8], promotes persistent colonization in mice [9].

Allelic diversity caused by high mutation rates and frequent recombination events is a striking property of *H. pylori* strains. Genetic fingerprints of individual strains obtained by multilocus sequence typing of housekeeping genes have indicated that clonal transmission is likely to occur, but is followed by a rapid adaptation to the new host, so that *H. pylori* isolates from different subjects are almost always unique [4]. On the other hand, while recombination events generating allelic diversity are frequent, genome changes involving gain or loss of genes seem to be rare [10]. Nevertheless, on the level of gene content, evidence has been presented that *H. pylori* is a species with an open pan-genome, in which each individual isolate contains a distinct set of non-core, or strain-specific, genes [6,11-13]. Comparative analysis of the first sequenced *H. pylori* genomes suggested that these strain-specific genes are often located in genomic regions that had previously been termed plasticity zones or plasticity regions, a designation originally used to describe a particular genetic locus with high variation between the first two *H. pylori* genome sequences [14]. However, with the availability of more sequencing data and more complete *H. pylori* genome sequences, it became clear that parts of the plasticity regions are usually organized as genomic islands that may be integrated in one of several different genetic loci. Furthermore, they generally contain complete sets of genes required to produce type IV secretion machineries, as well as genes encoding different DNA-processing proteins [11,15,16], suggesting that they are actually mobile genetic elements capable of horizontal gene transfer between bacterial cells, and that they might be best described as conjugative transposons or integrating conjugative elements (ICEs).

The actual plasticity of these islands partly derives from the fact that gene rearrangements, insertions or deletions may have occurred within them, but it is not clear whether they also carry variable passenger genes. Interestingly, intrahost variation among genes of the plasticity zones, including deletions in a type IV secretion system gene, has been found for sequential isolates obtained from a duodenal ulcer patient over a course of 10 years [17]. Although several candidate genes of these plasticity regions have been suggested as disease markers, e.g. *dupA* for duodenal ulcer [18,19], or *jhp950* for marginal zone B-cell MALT lymphoma [20], the functions of the plasticity zones are currently not well-understood.

To address the question of plasticity zone prevalence, and of their genetic diversity, we have performed a comparative analysis of these genome islands from a larger number of *H. pylori* genome sequences, including newly determined genome sequences of nine additional strains from different backgrounds. We show that these elements have a high prevalence throughout all populations, and that gene evolution within the elements is not congruent with the rest of the genomes. The wide variety of integration loci together with a conserved sequence motif at each integration site suggests an integration mechanism that depends on a short recognition motif in the DNA sequence only.

## Results

### Prevalence of plasticity regions in the *H. pylori* population

We have reported previously that *H. pylori* strain P12 contains three genome regions with similarity to the prototypical plasticity zones, but only one of them (PZ2) corresponds to the originally described locus, whereas the other two regions (PZ1 and PZ3) have a genetic organization typical for genome islands and contain genes for type IV secretion systems that might make them capable of self-transfer [11]. In comparison, the original two genome sequences (strains 26695 and J99) contain only truncated and highly rearranged portions of these genome islands (Additional file 1: Figure S1). As reported previously, the most conserved type IV secretion system genes fall into one of two distinct groups, which have been termed either *tfs3* and *tfs3a/b*[16], or *tfs3* and *tfs4*[11]. In accordance with Ref. [11], where conserved *tfs3* genes have been shown not to be more closely related to *tfs4* genes than to the respective *comB* genes encoding the type IV secretion system used for natural transformation, we consider *tfs3* and *tfs4* here as independent systems. Moreover, since there is evidence for horizontal gene transfer of the corresponding islands [11,16], but not for transposition within a strain, we propose to use the term integrating conjugative elements (ICE) and refer to individual islands as ICE*Hptfs3* or ICE*Hptfs4*, respectively. A comparison of different designations of the islands and associated type IV secretion systems is given in Table 1. To determine the occurrence of ICE*Hptfs3* and ICE*Hptfs4* elements in the *H. pylori* population and the degree of variation among them, we performed a comparative sequence analysis of these elements from 36 completely sequenced *H. pylori* genomes available in public databases (Table 2).

**Table 1:** Comparison of plasticity zone mobile genetic element and associated type IV secretion system (T4SS) designations

**Element designation used in this study**	**T4SS designation used in this study**	**Element designation used in [**[16]**]**	**T4SS designation used in [**[16]	**Element designation used in [**[11]
ICE*Hptfs3*	TFS3	TnPZ type 2	TFS3	PZ3
ICE*Hptfs4a*	TFS4a	TnPZ type 1b	TFS3b	PZ1
ICE*Hptfs4b*	TFS4b	TnPZ type 1	TFS3a	n.a.
ICE*Hptfs4c*	TFS4c	n.a.	n.a.	n.a.

n.a., not applicable.

**Table 2:** Properties of ICE elements in strains with complete genome sequences

**Strain**	**ICE type**	**Integration site (P12)**	**Pos. LJ**	**Pos. RJ**	**Size (kb)**^**14**^	**Complete T4SS?**
**52**	none					
**B38**	none^2^					
**F16**	none					
**HPAG1**	none					
**Sat464**	none					
**v225d**	none					
**26695**	ICE*Hptfs3*^3^	*hpp12*_981	1049829	473989	(16.0)	N
**26695**	ICE*Hptfs4a*/*4b*^3^	*hpp12*_1328^5^	1071598	464996	(18.3)	N
**35A**	ICE*Hptfs4a*	*hpp12*_92-91	359215	309788	(10.0)^15^	N
**51**	ICE*Hptfs3*/*4a*^3^	*hpp12*_999	none	1034232	(32.2)	N
**83**	ICE*Hptfs3*	*hpp12*_65	79085^12^	106931	(27.8)	N
**83**	ICE*Hptfs4b*	*hpp12*_1495	1522267	1503172^12^	(19.1)	N
**908, 2017, 2018**^**1**^	ICE*Hptfs4a*/*b*^4^	*hpp12*_995-979^6^	991801^12^	none	(14.6)	N
**B8**	ICE*Hptfs3*	*hpp12*_439-438	487322	526844	39.5^16^	Y
**B8**	ICE*Hptfs4a*	*hpp12*_1380-5S-rRNA^5,7^	528708^12^	452245	(37.0)	N
**Cuz20**	ICE*Hptfs4b*	*hpp12*_210-211	266516	227821	38.5	Y
**ELS37**	ICE*Hptfs3*	*hpp12*_511-512^8^	884907	838572	46.3	Y
**ELS37**	ICE*Hptfs4b*	*hpp12*_511-512^8^	838326	none	(2.0)	N
**F30**	ICE*Hptfs4a*	*hpp12*_92-91	1239533	1287710	(10.0)^15^	N
**F32**	ICE*Hptfs3*	*hpp12*_312-313	328469	1058181	(4.1 + 25.5)^17^	N
**F57**	ICE*Hptfs4a*	*hpp12*_92-91	152065	103732	(10.0)^15^	N
**F57**	ICE*Hptfs4b*	*hpp12*_259	323634	284294	39.3	Y
**G27**	ICE*Hptfs4b*	*hpp12*_1009-1010	1085072	1045702	39.4	Y
**Gambia 94/24**	ICE*Hptfs3*	*hpp12*_1508^5^	1473904	1521243	47.3	Y
**Gambia 94/24**	ICE*Hptfs4a*/*b*^4^	*hpp12*_994-5S-rRNA	1069322^12^	none	(35.2)	N
**HUP-B14**	ICE*Hptfs3*	*hpp12*_1365	1355656^13^	1355656^13^	(10.8)	N
**India 7**	ICE*Hptfs3*	*hpp12*_599	752074	798006	45.9	Y
**India 7**	ICE*Hptfs4a*	*hpp12*_1391-1528^10^	none	none	(7.3)	N
**J99**	ICE*Hptfs3*^3^	*hpp12*_444-445	1044878^13^	1044878^13^	(16.7)	N
**J99**	ICE*Hptfs4a*/*b*^4^	*hpp12*_994-5S-rRNA	none	none	(25.3)	N
**Lithuania 75**	ICE*Hptfs3*	*hpp12*_1508	1516637	none	(34.8)	N
**Lithuania 75**	ICE*Hptfs4c*	n.a. (plasmid integration)	3528^13^	3528^13^	(10.1)	N
**P12**	ICE*Hptfs3*	*hp*1354	1424780	1394778	(30.0)	N
**P12**	ICE*Hptfs4a*	*hp*0464	452023	492769	40.7	Y
**PeCan4**	ICE*Hptfs3*	*hpp12*_1528-1523^5^	1530039	1536824	(6.8)	N
**PeCan4**	ICE*Hptfs4a*	*hpp12*_1528-1523^5, 11^	1578142	1537082	41.1	Y
**PeCan18**	ICE*Hptfs3*	*hpp12*_440-439	1015120	1064481	49.4^16^	Y
**PeCan18**	ICE*Hptfs4a*	*hpp12*_994-5S-rRNA	1067535	none	(3.1)	N
**Puno120**	ICE*Hptfs3*	*hpp12*_994-5S-rRNA	1004976	none	(6.8 + 26.6)^18^	N
**Puno135**	ICE*Hptfs3*/*4b*^3^	*hpp12*_994-5S-rRNA	1014870	1059997	(45.1)	Y
**Shi112**	ICE*Hptfs3*	*hpp12*_226-225	281418	232869	48.6^16^	Y
**Shi112**	ICE*Hptfs4b*	*hpp12*_1380-5S-rRNA	1412827	1451480	38.7	Y
**Shi169**	ICE*Hptfs4b*	*hpp12*_211-210	240310	201136	39.2	Y
**Shi417**	ICE*Hptfs3*	*hpp12*_1510	1546576	1591512	(44.9)	Y
**Shi417**	ICE*Hptfs4b*	*hpp12*_1126-1125	1186887	1147709	39.2	Y
**Shi470**	ICE*Hptfs4b*	*hpp12*_495	874710	913872	39.2	Y
**SJM180**	ICE*Hptfs3*	*hpp12*_454-453	1413941	none	(23.2)	N
**SJM180**	ICE*Hptfs4a*	*hpp12*_1364-1365^9^	1371932	1416180^12^	(24.1)	N
**SNT49**	ICE*Hptfs4b*	*hpp12*_65	61216	100646	39.4	Y
**South Africa 7**	ICE*Hptfs4c*	*hpp12*_1366	1568674	1527381	41.3^16^	Y
**South Africa 7**	ICE*Hptfs4b*	*hpp12*_943-944	934499	973788	39.3	Y
**XZ274**	ICE*Hptfs4a*	*hpp12*_92-91	162178	111739	(10.0)^15^	N
**XZ274**	ICE*Hptfs4b*	*hpp12*_776	653446	612019	(41.4)^16^	Y

^1^Strains 908, 2017 and 2018 are sequential isolates from a single patient [17] and do not show major differences in their ICE*Hptfs4* sequences. However, note that GenBank entries EF195724.1, EF195725.1 and EF195726.1 describe ICE*Hptfs3* clusters in these strains [17] that are not present in the genome sequences.

^2^HELPY0971 is possibly a vestige of *hpp12*_1321/*pz7.*

^3^resulting from insertions of 2-3 genomic islands and subsequent rearrangements.

^4^containing ICE*HPtfs4a*-type genes close to the left junction, and ICE*Hptfs4b*-type genes close to the right junction.

^5^associated with genome rearrangement in comparison to strain P12.

^6^associated with deletion of *hpp12*_980 to *hpp12*_995 (5’) including one copy of 5S-23S-rRNA.

^7^associated with a recombination between the two 5S-23S-rRNA loci (including *hpp12*_1381-1384).

^8^partial duplication of both genes; ICE*Hptfs3* inserted into truncated ICE*Hptfs4b.*

^9^within a restriction-modification system inserted into this region.

^10^integrated together with a 0.9 kb fragment of ICE*Hptfs3* and a putative toxin-antitoxin system.

^11^integration of ICE*Hptfs4a* into remnant of ICE*Hptfs4b*, which is in turn integrated into truncated ICE*Hptfs3.*

^12^irregular integration, using internal AAGAATG motif.

^13^left and right junctions coincide due to irregular integration.

^14^numbers in parentheses indicate incomplete ICE elements.

^15^disrupted by a chromosomal inversion from *hpp12_*92 to *hpp12_*128.

^16^size of ICE increased by IS element insertion.

^17^interrupted by a chromosomal rearrangement between *hpp12*_312 and *hpp12*_1044 (including *babC* deletion).

^18^original integration probably in *hpp12*_994-5S-rRNA locus; from there, relocation of 26.6 kb fragment via internal AAGAATG motifs into *hpp12*_1510; 1.4 kb duplication (containing *xerT*) in both loci.

**Figure 1:**
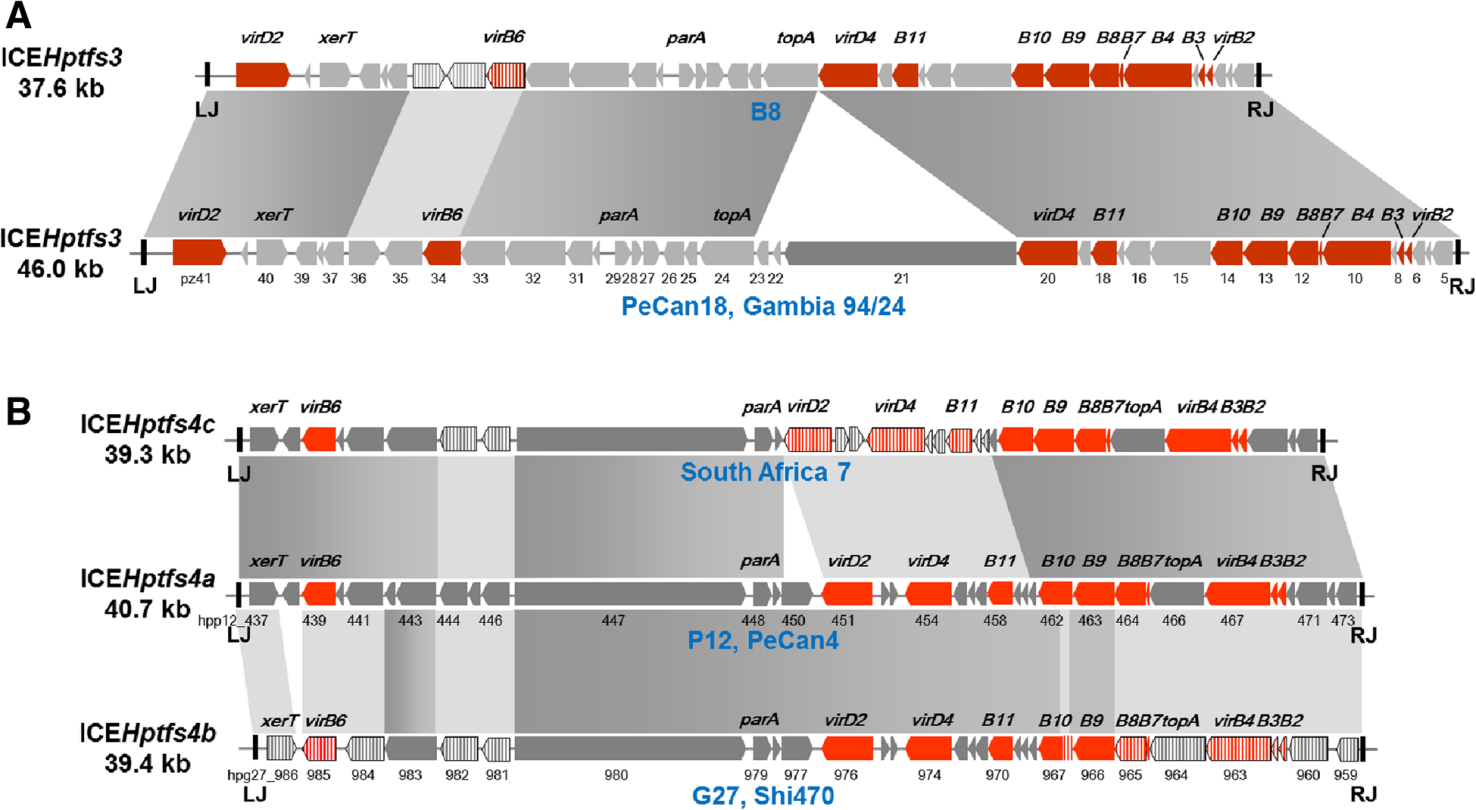
**Gene arrangement of prototypical ICE*Hptfs3 *(A) and ICE*Hptfs4 *(B) islands.** Genes encoding type IV secretion system components are drawn as red arrows, and other genes as grey arrows. Regions with high nucleotide sequence similarity are connected by dark grey bars, and regions with low to intermediate levels of similarity by light grey bars. Hatched arrows indicate orthologous, but clearly distinct gene variants. Typical sizes of the corresponding elements are indicated on the left. ICE*Hptfs3* elements differ by the presence or absence of *pz21*-*pz23* genes (according to the nomenclature of [15]) and by several distinct variants of the *pz34*, *pz35*, and/or *pz36* genes. However, variations within these two regions do not correlate with each other and were thus not considered for ICE*Hptfs3* subclassification. In contrast, ICE*Hptfs4* islands are further subclassified into ICE*Hptfs4a*, ICE*Hptfs4b* and ICE*Hptfs4c* groups according to the presence of orthologous gene variants. Note that the polymorphic genes *hpp12_446*/*hpg27_981* and *hpp12_444-445*/*hpg27_982* could not clearly be assigned to ICE*Hptfs4a* or ICE*Hptfs4b* and were thus not considered for classification of ICE*Hptfs4* subtypes. LJ, left junction; RJ, right junction.

We found that only 6 out of these 36 strains do not contain ICE*Hptfs3* or ICE*Hptfs4* islands or fragments thereof (Table 2). Among the remaining 30 strains, 19 harbour ICE*Hptfs3* islands, 6 of which seem to have complete gene sets, and 27 harbour ICE*Hptfs4* islands, 12 of which are complete. There are 3 strains with two different ICE*Hptfs4* elements, and 16 strains which have at least parts of both ICE*Hptfs3* and ICE*Hptfs4*. Three strains (strains 51, SJM180 and Puno135) contain hybrid arrangements of ICE*Hptfs3 and* ICE*Hptfs4* islands, but these seem to result from DNA rearrangements after integration of two independent genome islands (see below). Thus, each complete or truncated island can be assigned to either the ICE*Hptfs3* or the ICE*Hptfs4* type. Within the ICE*Hptfs3* group, two distinct variants can be discriminated, which differ by the presence (e.g., strain PeCan18) or absence (e.g., strain B8) of the *pz21*-*pz23* genes (Figure 1A). In contrast, three variants of ICE*Hptfs4*, defined by orthologous, but variant sets of genes at both ends of the genome islands, or in their central regions, can be distinguished and are termed here ICE*Hptfs4a*, ICE*Hptfs4b* and ICE*Hptfs4c*, respectively (Figure 1B; Table 1). The third subtype, ICE*Hptfs4c*, was only found in strain SouthAfrica7, which belongs to the hpAfrica2 population (see below), and as a plasmid-borne fragment in strain Lithuania75. Both types of genome island seem to vary considerably in size between strains (Table 2), but this is often due to small deletions within the islands or to insertion of IS elements; therefore, complete ICE*Hptfs3* islands have “standard” sizes of about 37.5, or 46 kb, depending on the presence of *pz21-23* orthologs, while complete ICE*Hptfs4a*, ICE*Hptfs4b* and ICE*Hptfs4c* usually comprise about 41, 39.5, and 39.5 kb, respectively (Figure 1A, B).

### Geographic distribution of ICE*Hptfs3* and ICE*Hptfs4* islands

It is well-established that *H. pylori* strains cluster into distinct populations according to their geographic origin when multilocus sequence typing using partial sequences of seven housekeeping genes is employed [21-23]. In contrast to this allelic variability, which suggests a common evolution of *H. pylori* and humans, consistent gene content profiles of individual populations could not be found, with the exception of one hypothetical gene (*jhp914*) present only in strains from the hpAfrica1 population [24]. Interestingly, comparison of gene content microarray data [24] with ICE*Hptfs4* composition suggests that most hpAfrica1 strains contain ICE*Hptfs4a* genes close to the left junctions and in the mid region (*jhp947*-*jhp951*; *hp1000*-*hp1006*; Additional file 1: Figure S1), but ICE*Hptfs4b* genes close to the right junctions (*jhp917*-*jhp924*; Additional file 1: Figure S1), while hpEurope strains variably contain these genes. Since there are only three hpAfrica1 strains among the 36 complete genome sequences analysed (strains 908, 2017 and 2018 were isolated from the same patient and are very similar), we decided to determine draft genome sequences of three further strains originating from Western Africa, as well as of six strains isolated in Europe, five of which had been tested positive for the presence of an ICE*Hptfs4a*-type or an ICE*Hptfs4b*-type *virB4* gene (data not shown). Sequence analysis revealed that all strains except one (196A) contain at least 37 kb of ICE*Hptfs3* and/or ICE*Hptfs4* sequences (Table 3).

**Table 3:** Properties of ICE elements identified in draft genome sequences

**Strain**	**Population**^**1**^	**ICE type**	**Integration site (P12)**	**Motif**	**Pos. LJ**^**4**^	**Size (kb)**^**5**^	**Complete T4SS (Y/N)**
**196A**	hpEurope	none				n.a.	
**166**	hpEurope	ICE*Hptfs4c*	*hpp12*_1518-1519	AAAGAATG	1613471	39.6	Y
**175**	hpEurope	ICE*Hptfs3*	*hpp12*_1366^3^	TAAGAATG	1440427	(10.8)	N
**175**	hpEurope	ICE*Hptfs4b*	*hpp12*_120	GAAGAATG	126992	(39.0)^6^	N
**175**	hpEurope	ICE*Hptfs4c*	*hpp12*_1510	TAAGAATG	1602176	39.3	Y
**328**	hpEurope	ICE*Hptfs4a*	*hpg27*_335	AAAGAATA	366213	(2.3)	N
**328**	hpEurope	ICE*Hptfs4b*	*hpp12*_1365	AAAGAATG	1436629	40.2	Y
**ATCC43526**	hpEurope	ICE*Hptfs3/*4a^2^	*hpp12*_1508	TAAGAATG	1598758	(47.5)	N
**ATCC43526**	hpEurope	ICE*Hptfs4a*	*hpp12*_189-188	TAAGAATG	191853	(22.6)	N
**P1**	hpEurope	ICE*Hptfs3*	*hpp12*_746-745	AAACAATA	800162	(13.3)	N
**P1**	hpEurope	ICE*Hptfs4b*	*hpp12*_1366	TAAGAATG	1439080	39.4	Y
**1_17C**	hpAfrica1	ICE*Hptfs4a*/*b*	*hpp12*_994-5S-rRNA		1054197	(37.6)	N
**6_17A**	hpAfrica1	ICE*Hptfs4a*/*b*	*hpp12*_994-5S-rRNA		1054197	(37.7)	N
**6_28C**	hpAfrica1	ICE*Hptfs4a*	*hpp12*_994-5S-rRNA		1054197	(1.6)	N
**6_28C**	hpAfrica1	ICE*Hptfs4b*	*hpp12*_438	AAAGAATG	453993	(35.5)	N

^1^inferred from the Neighbor-joining tree shown in Figure 2.

^2^resulting from insertion of two genome islands and rearrangements associated with IS element insertion and two copies of *pz21*/*hpp12*_447-like genes.

^3^associated with a genome rearrangement between *hpp12*_1366 and *hpp12*_1298.

^4^genomic position of AAGAATG motif in strain P12.

^5^numbers in parentheses indicate incomplete ICE elements.

^6^contains 28 kb of prophage-related sequences.

**Figure 2:**
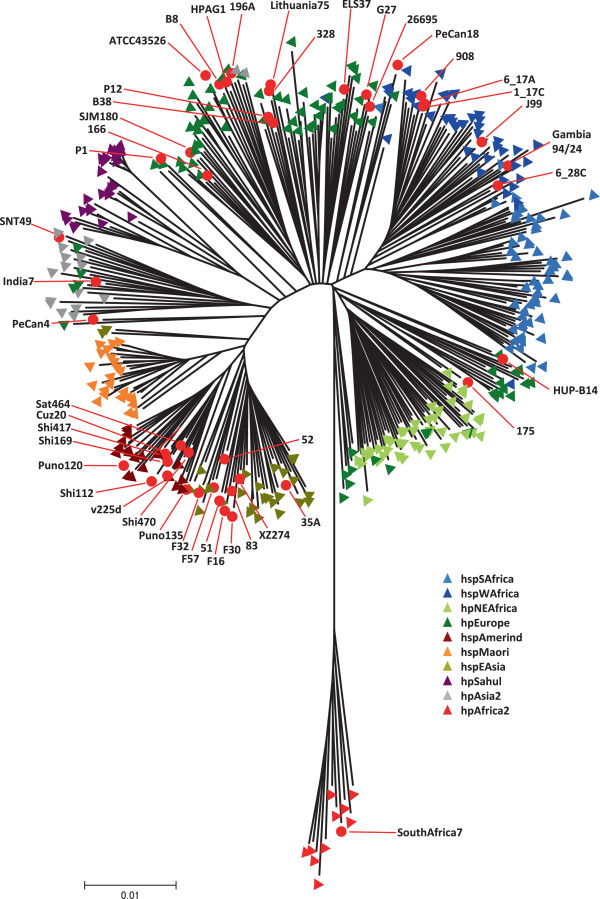
**Phylogeography of the analysed strains.** The Neighbor-joining tree was calculated with concatenated MLST sequences from 345 reference strains from the *H. pylori* MLST database ( http://pubmlst.org/helicobacter/) and from all strains analysed in this study. MLST database phylogeography assignments are indicated by coloured triangles, and locations of sequenced strains are indicated by red dots.

**Figure 3:**
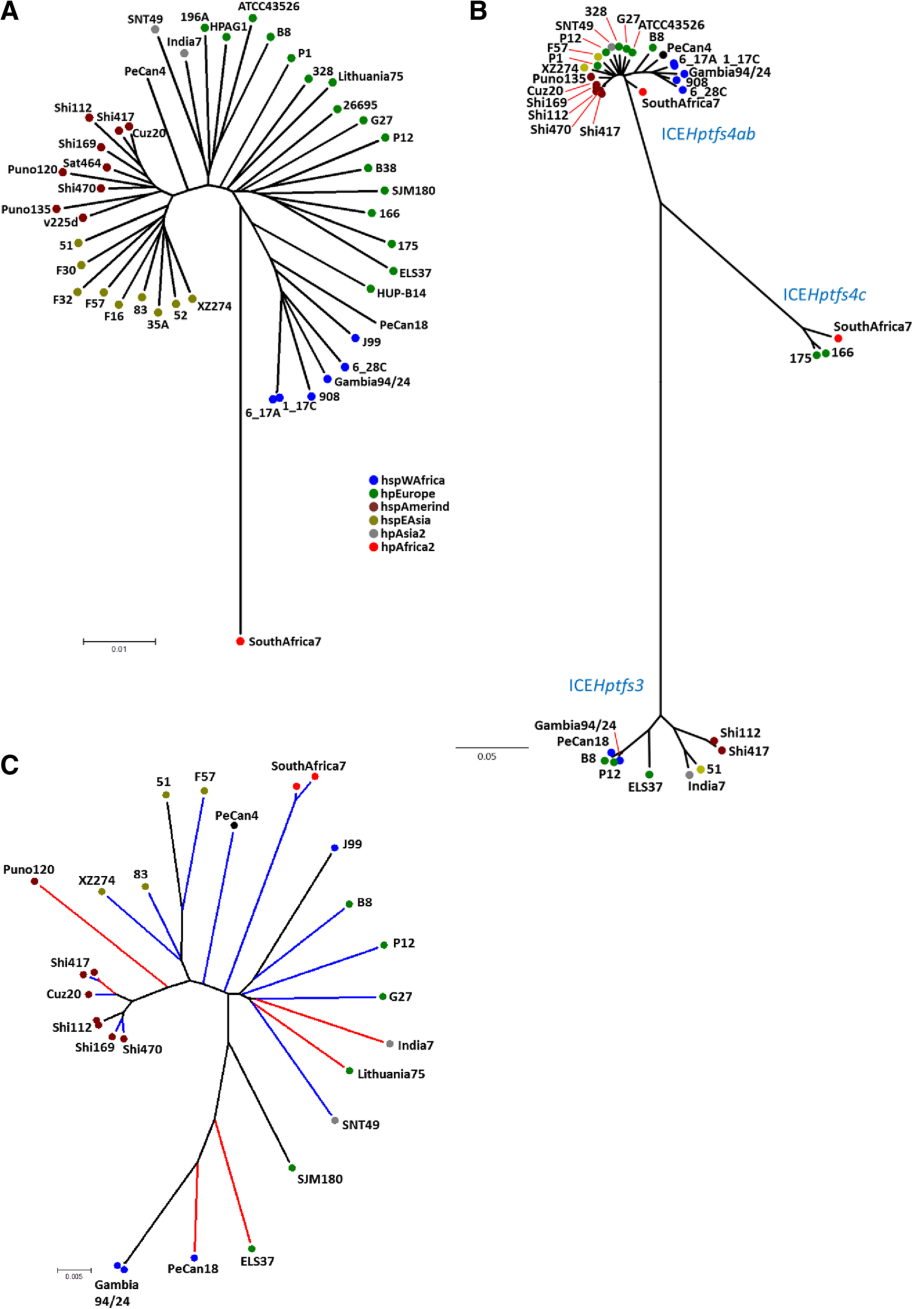
**Neighbor-joining analysis of type IV secretion system gene sequences. (A)** Phylogenetic tree calculated with MLST sequences for fully sequenced strains only, with phylogeography assignments based on the Neighbor-joining tree shown in Figure 2. Note that unequivocal classification of strains PeCan4 and PeCan18 was not possible. **(B)** Phylogenetic tree calculated from concatenated *virB9*, *virB11* and *virD4* ortholog sequences of all ICE*Hptfs3* and ICE*Hptfs4* islands. **(C)** Neighbor-joining tree calculated from DNA sequences of methylase/helicase (*hpp12*_447/*pz21*) orthologs. Orthologs associated with ICE*Hptfs3* elements are marked by blue branch lines, and orthologs associated with ICE*Hptfs4* elements by red branch lines. Black lines indicate hybrid elements or the presence of two different elements in the same strain. Colouring of individual strains by phylogeographic origin is shown according to the tree in Figure 2.

To examine possible variations in plasticity zone distribution among phylogeographic groups, we first constructed a phylogenetic tree based on MLST gene sequences, using all 36 fully sequenced strains, the nine strains sequenced in this study, and 345 reference strains from the MLST database (Figure 2). No correlation between phylogeographic groups and the presence or absence of either ICE*Hptfs3* or ICE*Hptfs4* could be found. However, all hpAfrica1 strains contain truncated versions of ICE*Hptfs4b* or of an ICE*Hptfs4a*/*b* variant similar to the hpAfrica1 strains mentioned above (Tables 2 and 3). We then calculated Neighbor-joining phylogenetic trees using conserved ICE*Hptfs3* or ICE*Hptfs4* gene sequences (concatenated *virB9*, *virB11* and *virD4* sequences) and compared them with an MLST-derived tree (Figure 3A, B). Interestingly, ICE*Hptfs4ab* genes clustered in a similar way as housekeeping gene sequences did, except for a much closer relationship of these genes than of housekeeping genes between hpAfrica2 strain SouthAfrica7 and other populations (Figure 3B; Additional file 2: Figure S2). In contrast, ICE*Hptfs3* sequences formed at least three strongly divergent clades that were not congruent with the MLST population structure. These clades seem to correspond to (1) the hspAmerind population; (2) a mixture of hspEAsia and hpAsia2 populations; and (3) a mixture of hpEurope and hpAfrica1 populations (Figure 3B; Additional file 2: Figure S2). However, the number of ICE*Hptfs3*-positive strains analysed may be too low to definitely draw conclusions from this observation.

### Identification of conserved and ICE type-specific genes

Since both ICE*Hptfs3* and ICE*Hptfs4* islands contain genes for complete type IV secretion systems and may coexist in a single strain, an open question is whether individual genes or groups of genes from one type of island have the capacity to complement deficiencies in the other. Sequence comparisons showed that each of the type IV secretion apparatus components is clearly distinguishable between the different types (and partly between subtypes) of islands, with amino acid sequence similarities ranging from 40% to 80% (Table 4). This is also true for putative DNA processing or segregation proteins such as XerT, ParA, TopA or VirD2 (but not for the putative methylase/helicase PZ21 (OrfQ)/HPP12_447; see below), suggesting that the individual secretion systems might be sufficiently divergent to be incompatible.

**Table 4:** **Amino acid similarities and identities between ICE *Hptfs4a *-encoded proteins and proteins from ICE** ***Hptfs3 *** **and ICE** ***Hptfs4b/c *** **islands**

**Gene P12**	**Size (aa)**	**Identity/similarity ICE** ***Hptfs4b***^**1**^	**Identity/similarity ICE** ***Hptfs4c***^**1**^	**Orthologous gene on ICE** ***Hptfs3***	**Identity/similarity ICE** ***Hptfs3***^**1**^	**Putative function**
*hpp12*_437	357	56/73	98/98	*hpb8*_521/pz40	**63/76**	XerT
*hpp12*_438	227	missing	95/97	*hpb8*_524/pz37	**77/83**	
*hpp12*_439	432	**32/49**	93/95	*hpb8*_527/pz34	**23/42**^**2**^	VirB6
*hpp12*_440	92	missing	93/96	missing	-	
*hpp12*_441	466	**40/60**	96/97	*hpb8*_526/pz35	**23/46**^**2**^	
*hpp12*_442/443	737	94/95	94/95	*hpb8*_543/pz15	**32/50**	
*hpp12*_444/445	464	**28/46**	97/99^3^	*hpb8*_529/pz32	**26/53**^**4**^	
*hpp12*_446	340	**28/43**	95/97^3^	*hpb8*_530/pz31	**30/47**	
*hpp12*_447	2808	94/96	92/95	pz21	89/93	
*hpp12*_448	218	98/99	97/99	*hpb8*_532/pz29	**67/81**	ParA
*hpp12*_449	94	98/100	94/94	*hpb8*_533/pz28	**37/69**	
*hpp12*_450	392	92/96	missing	missing	-	
*hpp12*_451	637	93/95	**35/51**	*hpb8*_519,517/pz41	**35/56**	VirD2
*hpp12*_452	104	97/98	n.d.^5^	missing	-	
*hpp12*_453	93	98/100	n.d.^5^	missing	-	
*hpp12*_454	575	98/99	**62/77**	*hpb8*_538/pz20	**50/66**	VirD4
*hpp12*_455	170	98/98	**46/60**^**6**^	*hpb8*_538/pz20^6^	**32/50**	
*hpp12*_456	96	97/98	n.d.^5^	missing	-	
*hpp12*_457	151	97/97	**35/51**	missing	-	
*hpp12*_458	313	99/99	**58/74**	*hpb8*_540/pz18	**42/64**	VirB11
*hpp12*_459	99	98/100	n.d.^5^	missing	-	
*hpp12*_460	87	93/96	n.d.^5^	missing	-	
*hpp12*_461	97	97/97	91/93	missing	-	
*hpp12*_462	421	**80/84**	92/95	*hpb8*_544/pz14	**53/69**	VirB10
*hpp12*_463	510	97/98	94/97	*hpb8*_545/pz13	**47/66**	VirB9
*hpp12*_464	389	**55/73**	98/99	*hpb8*_546/pz12	**38/62**	VirB8
*hpp12*_465	38	**55/75**	55/75	*hpb8*_547/pz11	**44/58**	VirB7
*hpp12*_466	677	**45/62**	94/97	*hpb8*_537/pz24	**45/61**	TopA
*hpp12*_467	807	**44/63**	96/97	*hpb8*_548/pz10	**38/58**	VirB4
*hpp12*_468	88	**54/75**	95/97	*hpb8*_550/pz8	**39/58**	VirB3
*hpp12*_469	100	**42/63**	93/97	*hpb8*_551/pz7	**30/45**	VirB2
*hpp12*_470/471	508	**34/54**	94/96	*hpb8*_528/pz33	**35/51**	
*hpp12*_472	97	missing	90/93	missing	-	
*hpp12*_473	259	**34/57**	92/93	*hpb8*_554/pz5	**37/63**	

^1^numbers printed in normal face correspond to >90% identity (identical genes), and numbers in bold face to 40-85% similarity.

^2^genes *hpb8*_526 and *pz35*, as well as *hpb8*_527 and *pz34* share only 61/73% and 54/70% identity/similarity, respectively, to each other, but are equally similar to *hpp12*_441 and *hpp12*_439, respectively.

^3^some ICE*Hptfs4c* islands contain the ICE*Hptfs4b* versions with lower similarities in these sites.

^4^similarities confined to short regions only.

^5^no significant similarity detectable, but gene with similar size and orientation present.

^6^ICE*Hptfs4c* and ICE*Hptfs3* islands contain fusions of *hpp12*_454 and *hpp12*_455.

To define further common ICE gene products and to identify ICE-type-specific genes, we performed similarity searches with all other amino acid sequences as well. The results show that nine further, hypothetical ICE*Hptfs4a* genes have similar counterparts in ICE*Hptfs3*-type islands (Table 4). Interestingly, orthologs of the conserved hypothetical genes *hpb8*_524 or *hpp12*_438 are present in ICE*Hptfs3*, ICE*Hptfs4a* and ICE*Hptfs4c* islands, but absent from ICE*Hptfs4b* islands. Because of their sequence similarities, we speculate that these hypothetical genes have additional conserved functions for genome island maintenance and/or transfer. In contrast, genes that are specific for either type of genome island might be cargo proteins of the respective mobile genetic elements, fulfilling more specific roles. Such specific genes for ICE*Hptfs4* islands are *hpp12*_440 (present only on ICE*Hptfs4a* and ICE*Hptfs4c* islands), *hpp12*_450/*hpg27*_977 (which is specifically absent in ICE*Hptfs4c* islands), *hpp12*_452, *hpp12*_453, *hpp12*_456, *hpp12*_459-461, and *hpp12*_472 (Table 4). Specific genes of ICE*Hptfs3* islands include *hpb8*_522, *hpb8*_523, *hpb8*_525, *hpb8*_531, *hpb8*_534, *hpb8*_535, *hpb8*_539, *hpb8*_541, *hpb8*_542, *hpb8*_549, *hpb8*_552, *pz22* and *pz23*. Interestingly, ICE*Hptfs3* islands in some strains have insertions of specific genes encoding Fic domain-containing or JHP940-like proteins (Additional file 3: Figure S3).

The putative DNA methylase/helicase gene *pz21***(***orfQ*)/*hpp12*_447 may be found associated with either ICE*Hptfs3* or ICE*Hptfs4* islands. In striking contrast to the above-mentioned divergence between orthologous ICE*Hptfs3* and ICE*Hptfs4* genes, the methylase/helicase orthologs present on ICE*Hptfs3* (e.g., *pz21*) and on ICE*Hptfs4a/b/c* islands (e.g., *hpp12*_447) are highly conserved (90-98% similarity), indicating an evolutionary pressure for this gene which is distinct from other genes on the genome islands. A Neighbor-joining tree of *pz21*/*hpp12*_447 orthologs shows a certain clustering according to geographic origin, but this clustering is clearly independent of gene association with either ICE*Hptfs3* or ICE*Hptfs4* (Figure 3C). Indeed, in cases where both ICE*Hptfs3* and ICE*Hptfs4* methylase/helicase orthologs are present in a single strain (Shi112, Shi417, Gambia94/24), these orthologs are always more similar to each other than to ICE*Hptfs3* or ICE*Hptfs4* orthologs of geographically related strains, and even more similar than two ICE*Hptfs4* methylase/helicase orthologs present in a single strain (SouthAfrica7) are to each other (Figure 3C). Because of these high sequence similarities, homologous recombination between ICE*Hptfs3* and ICE*Hptfs4* methylase/helicase orthologs is possible. By analysing the gene arrangements of the hybrid ICE*Hptfs3*-ICE*Hptfs4* elements mentioned above, we could identify situations where such recombination events seem to have occurred indeed after integration of one ICE element into another (Additional file 4: Figure S4).

### Analysis of ICE integration sites

**Figure 4:**
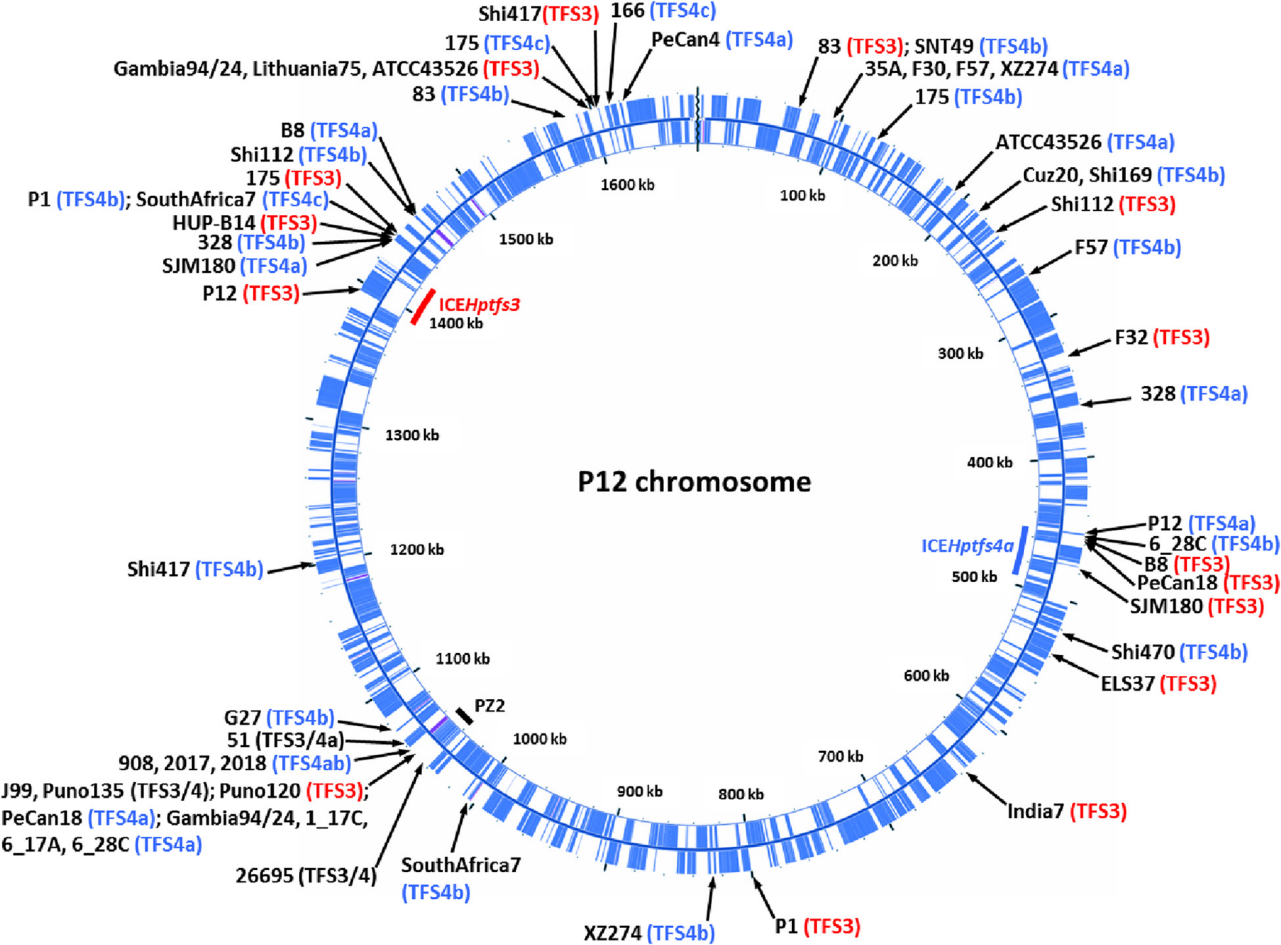
**Integration sites of all ICE*Hptfs3 *and ICE*Hptfs4 *islands mapped onto the genome of strain P12.** Positions of these elements as well as of plasticity zone 2 (PZ2) in the genome of P12 are shown within the circle. Each arrow indicates an individual ICE*Hptfs3* and/or ICE*Hptfs4* integration site. Note that the integration sites shown for strains where one island is integrated into another are not indicative of their genomic location in comparison to the main genome (for example, ICE*Hptfs3* of strain PeCan18 is inserted into a ICE*Hptfs4a* fragment and therefore shown at 456 kb, but the ICE*Hptfs4a* fragment is in fact integrated in the PZ2 region at 1059 kb in this strain).

Originally, the plasticity zone was found located at a distinct position within *H. pylori* genomes (i.e., between the *ftsZ* gene (*hp0979*) and one copy of the 5S-23S rRNA genes) [14]. However, analysis of strain P12, Shi470 and G27 genome sequences showed that ICE*Hptfs3* and ICE*Hptfs4* elements are able to integrate as well into different genomic locations, in a manner similar to conjugative transposons or genome islands [11,16]. To examine further variations in integration sites, we compared the sequences of ICE integration sites and duplicated junction motifs in all genome sequences with recognizable left and/or right ICE*Hptfs3* and ICE*Hptfs4* junctions. In addition to 12 different sites described previously [16], we identified further 28 chromosomal sites and one plasmid site where complete or partial ICE*Hptfs3* or ICE*Hptfs4* elements can be integrated (Tables 2 and 3; Figure 4). Although these integration sites cluster in certain genome regions, such as the originally identified ICE integration locus (plasticity zone 2 in P12), the left border region of ICE*Hptfs4a*, or a locus containing several restriction-modification system genes (*hpp12*_1364-1366), there is no obvious general preference for ICE integration. We also did not observe different patterns of ICE*Hptfs3* versus ICE*Hptfs4* integration sites; in fact, some integration sites are used by either ICE*Hptfs3* or ICE*Hptfs4* (Figure 4).

**Figure 5:**
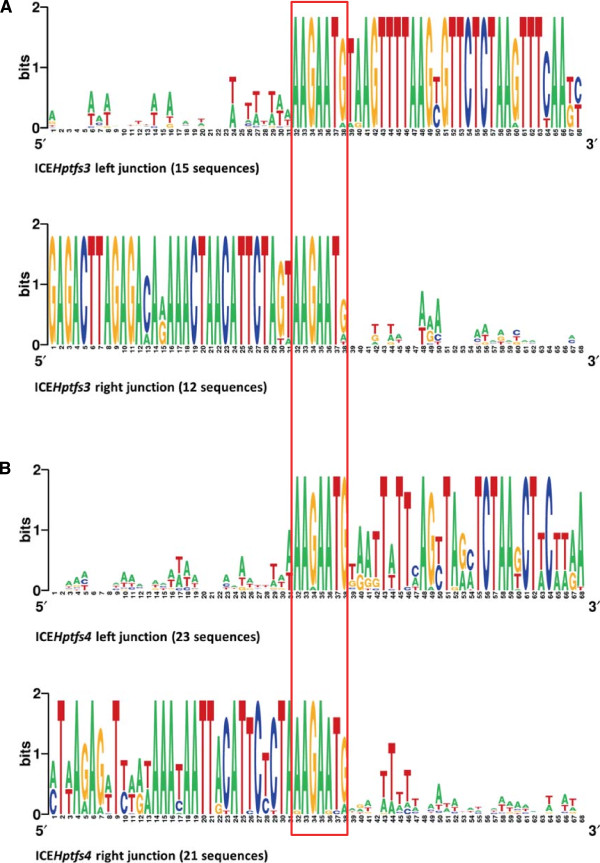
**Comparative analysis of integration sites.** Sequence logos for nucleotide sequences around ICE*Hptfs3***(A)** or ICE*Hptfs4***(B)** integration sites were generated using Weblogo [43]. The level of sequence conservation is indicated by the height of the letters (with a maximum of 2 bits at each position).

All islands with detectable junctions contained the conserved sequence motif AAGAATG [11,16], and this motif is always present in the corresponding empty sites of PZ-free strains (albeit sometimes mutated), suggesting that it represents a minimal requirement for integration of ICE*Hptfs3* and ICE*Hptfs4* elements. To determine whether additional sequences are required to form an integration site, we compared the sequences of the flanking regions of ICE*Hptfs3* and ICE*Hptfs4* separately (Figure 5; Additional file 5: Figure S5). There is a certain preference for A or T close to the left junctions of both ICE*Hptfs3* and ICE*Hptfs4* islands (-1 to -3 or -1 to -6), but the alignment revealed no significant consensus sequences otherwise. However, there seems to be a stronger preference of A at the -1 position (resulting in AAAGAATG motifs) in ICE*Hptfs4* than in ICE*Hptfs3* islands. Furthermore, the low prevalence of the last G at the right junctions of ICE*Hptfs3* islands may even suggest that only six bases (AAGAAT) are used by ICE*Hptfs3* islands.

### Identification of a unique ICE*Hptfs4* variant in the hpAfrica1 population

**Figure 6:**
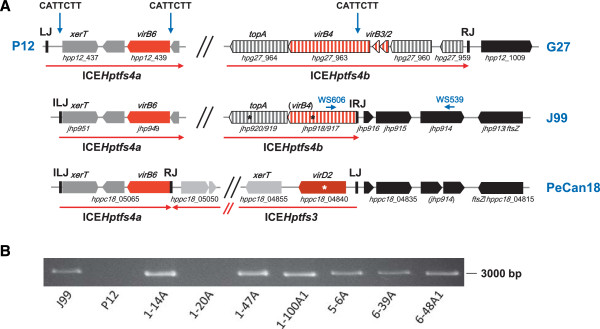
**A truncated version of ICE*Hptfs4 *in hspWAfrica strains. (A)** Most hspWAfrica strains (exemplified here by J99) have an ICE*Hptfs4* variant composed of ICE*Hptfs4a* genes (compared here with P12) close to the left junction and ICE*Hptfs4b* genes (compared here with G27) close to the right junction of the island. In these strains, the left part of the island is shortened by 350 bp at a CATTCTT motif upstream of *xerT*, and the right part by approximately 3850 bp at a CATTCTT motif within ICE*Hptfs4b virB4*, generating irregular left and right junctions (ILJ and IRJ). In strain PeCan18, the ICE*Hptfs4a* fragment has probably been integrated in a similar manner, using irregular integration at the same chromosomal position, but the majority of ICE*Hptfs4a* seems to have been deleted subsequently by (regular) integration of an ICE*Hptfs3* at the same internal *virB4* motif and another internal CATTCTT motif upstream of ICE*Hptfs4a virB6*. Gene colouring is as in Figure 1, and asterisks denote frameshift or nonsense mutations **(B)** PCR analysis of the ICE*Hptfs4b* right junction in *H. pylori* strains from Nigeria. PCR was performed from chromosomal DNA of the indicated strains with primers WS606 and WS539 (see Figure 6A).

Since deletions of single genes or different sets of genes are frequent for both ICE*Hptfs3* and ICE*Hptfs4* islands (Table 2), we checked whether these occur randomly or at conserved sites. Deletions found within ICE*Hptfs3* variants range from small deletions (*pz26* and *pz27*) to loss of major parts of the island (Additional file 3: Figure S3A), and mostly seem to occur at random positions and without conserved sequence motifs (data not shown). However, we also identified several cases where ICE*Hptfs3* truncation sites are flanked by AAGAATG motifs, suggesting that recombination events similar to ICE integration resulted in some deletions (Additional file 3: Figure S3A). For ICE*Hptfs4* islands, we found certain deletions that are more frequent. For example, four hspEAsia strains (35A, F30, F57, XZ274) have identical truncations of their ICE*Hptfs4a* islands (Additional file 3: Figure S3B). These elements also have identical integration sites (Figure 4) and are accompanied by a common genome rearrangement [25], suggesting that the observed truncations reflect the situation in a common ancestor of all four strains. In fact, these truncated versions are the only ICE*Hptfs4a* remnants that we found in hspEAsia or hspAmerind strains; all other complete or truncated variants in these populations are of the ICE*Hptfs4b* type. A second common truncation was found in all hspWAfrica strains (908/2017/2018, Gambia94/24, 1_17C, 6_17A, 6_28C) and involved a loss of several genes close to the right junctions of their ICE*Hptfs4b* or ICE*Hptfs4a*/*b* islands, including the 5’ regions of the respective *virB4* genes (Additional file 3: Figure S3B). The same deletion occurs in hspWAfrica strain J99, where the corresponding *virB4* gene (*jhp917*/*918*) is also known as *dupA*[18]. All these ICE*Hptfs4b* islands have their right junctions deleted and are furthermore inserted at the same genome position (Tables 2 and 3), flanked on the truncation site by *jhp916*, *jhp915* and *jhp914* orthologs (Figure 6A). A closer inspection of the right border revealed that truncations have occurred at a CATTCTT (or AAGAATG on the reverse strand) motif which is conserved in the *virB4* genes of ICE*Hptfs4b* (but not ICE*Hptfs4a*) islands. Interestingly, those ICE*Hptfs4b* variants which contain ICE*Hptfs4a* genes close to their left borders, all have another small truncation of about 300 bp at their left junctions, which also has occurred at a conserved CATTCTT motif upstream of the *xerT* gene (Additional file 3: Figure S3B), indicating that these islands have integrated in an irregular fashion, producing irregular left junctions (ILJ) and irregular right junctions (IRJ; Figure 6A). Since the nearby *jhp914* gene has previously been reported to be specifically present in the hpAfrica1 population [24], we asked whether this truncated right border might be a general signature of hpAfrica1 strains. To test this hypothesis, we performed a BLAST search of draft genome sequences with a 260 bp query sequence spanning the right border of J99 (including the IRJ). Of 78 retrieved draft genome sequences having the same IRJ, 64 also contained the *jhp914* gene (data not shown). Furthermore, we checked a panel of *H. pylori* strains isolated in Nigeria for the presence of the irregular ICE*Hptfs4b* right border (Figure 6B). PCR analysis with primers specific to *virB4* and *jhp914*, respectively (Figure 6A), confirmed that 14 out of 19 strains from this population were positive for a similar gene arrangement in this locus and thus for an IRJ (Figure 6B, and data not shown).

## Discussion

The unusual genetic heterogeneity of *H. pylori* has been well-documented in terms of allelic diversity, establishing it as a species with a very high population recombination rate, and allowing for different populations from different geographic regions to be identified [4]. MLST analysis of these populations has revealed important insights into the coevolution of *H. pylori* and humans, and into migration events of human populations, but relatively little is known about bacterial population-specific properties on a genomic level. Striking differences in the presence or absence of putative host interaction genes have been reported for East Asian *H. pylori* strains in comparison to European strains [12], and many divergent genes were found to evolve under positive selection between East Asian and non-Asian strains [12,26]. Previous comparative analysis of a small number of *H. pylori* genome sequences indicated that many strain-specific genes are located either at potential genome rearrangement sites or within the plasticity zones [11]. However, for those plasticity zone regions that are organized in ICE*Hptfs3* or ICE*Hptfs4* islands as described here, identification of further novel genes seems unlikely. Instead, the gene content of a given type of ICE*Hptfs3* or ICE*Hptfs4* island is, apart from the variable presence of JHP940- or Fic domain protein-encoding genes, highly conserved, strongly suggesting that these elements are autonomous elements with fixed contents rather than true regions of genome plasticity. Nevertheless, partial truncations, insertions of restriction-modification systems, IS elements or even distinct genome islands, and associated rearrangements [25] are frequent within both types of ICE and result in a considerable amount of variation. Rearrangements between ICE*Hptfs3* and ICE*Hptfs4* elements may be facilitated by recombination events within *pz21*/*hpp12*_447 (methylase/helicase) orthologs present on both types of islands. Apart from that, ICE*Hptfs3* and ICE*Hptfs4* islands are clearly distinct and do not seem to exchange individual genes. The fact that *pz21*/*hpp12*_447 orthologs are the only genes with high similarity between ICE*Hptfs3* and ICE*Hptfs4* elements, indicates that these orthologs are either frequently exchanged between both types of island, or that they are subject to strong selective pressures.

Interestingly, certain regions of both ICE*Hptfs3* and ICE*Hptfs4* islands are much more variable than others. For instance, we were able to identify 3, 5, and 4 distinct clades, respectively, for the *pz34*, *pz35* and *pz36* orthologs on ICE*Hptfs3* elements (data not shown), whereas all other ICE*Hptfs3* genes are more conserved. However, similar to the variability of *hpp12*_444/445 and *hpp12*_446 orthologs among ICE*Hptfs4* islands, where two clades each can be distinguished (data not shown), no clear correlation of these different clades with individual geographic groups could be found. Likewise, the three different subtypes of ICE*Hptfs4* islands which are characterized by orthologous, but distinct sets of genes, do not seem to be restricted to certain geographic groups. We also performed a preliminary analysis of two further hpAfrica2 strain genome sequences [27] and one hpSahul strain genome sequence [13] that were published after completion of our comparative analysis. Both hpAfrica2 strains contain one full-length ICE*Hptfs4b* element, and the hpSahul strain harbours a full-length ICE*Hptfs4b* and a partial ICE*Hptfs3* element (data not shown), which further supports the notion that these elements are present in all phylogeographic groups. The modular structure of ICE*Hptfs4* islands indicates that parts of these elements can easily be exchanged, and that all variants may coexist in a given *H. pylori* population. Indeed, ICE*Hptfs4a*, *b* and *c* islands all have some common genes which may be used for exchange of modules. However, it is striking that all members of ICE*Hptfs4b* subtypes consistently lack *hpp12*_438 orthologs and that hybrid elements between different ICE*Hptfs4* subtypes do not occur. An exception is the combination of ICE*Hptfs4a* (left) with ICE*Hptfs4b* (right), which seems to occur in hpAfrica1 strains only, and always in a truncated version. These restrictions on modular exchange suggest that there is a selective pressure on maintenance of cognate left and right ICE*Hptfs4* ends, for example by an inability of hybrid elements to be excised and/or transferred. The presence of ICE*Hptfs3*-like islands in other *Helicobacter* species, such as *H. cetorum*[16,28] and *H. suis*[29], indicates that these elements were acquired a long time ago (i.e., before the *cag* pathogenicity island, which is absent in hpAfrica2 strains and was acquired more than 60000 years ago [30]). Whereas microdiversity within *cag* pathogenicity island genes correlates with microdiversity in housekeeping genes, this is not the case for ICE*Hptfs3* or ICE*Hptfs4* genes, which shows again that these islands are subject to more frequent horizontal gene transfer.

Horizontal gene transfer of typical ICEs involves several steps [31]: first, the element is usually excised from the chromosome by a recombinase to generate a circular intermediate; second, this circular form is transferred from the donor to a recipient cell by conjugation; and third, the ICE integrates into the recipient cell chromosome via site-specific or unspecific recombination. In the case of ICE*Hptfs4*, the first step is dependent on the XerT recombinase [11], and the second on the VirD2 relaxase [32], both of which are encoded on the ICE. It is likely, but has not been shown yet, that the ICE-encoded type IV secretion system is responsible for the conjugative transfer process. It is also currently unclear whether the XerT recombinase catalyzes integration of the ICE into the recipient cell chromosome as well. An interesting finding of this study was the presumptive minimal requirement for integration of both ICE*Hptfs3* and ICE*Hptfs4* islands, the sequence motif AAGAATG (or possibly AAGAAT for ICE*Hptfs3*), as suspected previously [11,16]. Thus, the total number of possible insertion sites might be limited only by the number of these motifs in intergenic regions or in non-essential genes. In total, we identified more than 40 different integration sites, but the total number of possible integration sites might be significantly higher, given that AAGAATG sequences are found approximately 550 times within individual *H. pylori* genomes (data not shown). Many well-characterized ICEs integrate into a unique position in the host cell genome (the primary attachment site), often in the 3’ regions of tRNA loci [31]. In the absence of primary attachment sites, these elements are sometimes capable of integrating into secondary sites with much less specificity, but this may result in ICE immobility or even toxicity for the host cell [33]. In contrast, other ICE-like elements, which are often termed conjugative transposons, have very low integration site specificities, with as many as 100,000 possible integration sites in a given host strain [34,35]. In this regard, ICE*Hptfs3* and ICE*Hptfs4* seem to integrate with an intermediate specificity, but still with the potential to insert into coding regions and thereby to disrupt essential genes. Possible integration sites are also located on the ICE elements themselves, and we found several cases where one ICE is integrated into another. We could also identify situations where these internal sites were used for irregular ICE integration, associated with truncation of the left and/or right ICE ends, and possibly an incapability of these elements to excise.

Finally, despite the presence of genes encoding host interaction factors such as JHP940 [36], or correlated with disease outcome, such as *dupA*[18], the (potentially different) functions of ICE*Hptfs3* and ICE*Hptfs4* islands are currently unclear. In our analysis, a total of 18 strains were positive for *dupA* (the ICE*Hptfs4b virB4* gene), and 12 additional strains were found positive for ICE*Hptfs4a* or ICE*Hptfs4c virB4* genes, which are likely to have the same functions. Because of this, and since not all of these strains have complete ICEs or even complete type IV secretion systems, testing for the presence of the *dupA* gene alone, and correlations of *dupA* with pathology is probably not useful. It has been shown that a more complete analysis of type IV secretion system genes is more significant as a virulence marker [19]. Therefore, future correlation studies should determine the presence of the complete set of genes.

## Conclusions

Taken together, our comparative analysis reinforces the notion that major parts of the *H. pylori* plasticity zones described earlier should in fact be considered as mobile genetic elements with conserved gene content, rather than regions of genome plasticity. Although horizontal gene transfer of complete ICE*Hptfs3* or ICE*Hptfs4* elements remains to be demonstrated experimentally, the number of different integration sites indicates a considerable mobility, possibly also within individual *H. pylori* genomes. In this regard, these elements differ from the *cag* pathogenicity island, for which only one integration site is known (although rearrangements may occur). The high prevalence and wide distribution of these ICEs throughout all *H. pylori* populations suggest that they might provide an as yet unknown fitness benefit to their hosts.

## Methods

### Draft genome sequencing of H. pylori strains

To select *H. pylori* strains for draft genome sequencing, chromosomal DNA was prepared from a panel of laboratory strains or of clinical isolates, using a QIAamp DNA mini kit, and analysed by PCR with primer pair DupA-WXF (5′-GATATACCATGGATGAGTTCYRTAYTAACAGAC-3′) and JHP0919R2 (5′-GCCCACCAGTTGCAAAAACAAATGAAC-3′) [37], or with primer pair WS393 (5′-TATGGTATCAGGGCATACC) and WS394 (5′-GTTCTTTGAGATACTCAGG-3′) for the presence of ICE*Hptfs4b* or ICE*Hptfs4a virB4*, respectively. Based on this analysis, we selected 3 *virB4*-positive strains isolated in Western Africa, 5 *virB4*-positive strains isolated in Europe, and one *virB4*-negative strain isolated in Europe for genome sequencing.

Whole genomic DNA was isolated from bacteria that were subjected to minimal passage, using Qiagen Genomic‒tip 100/G columns and the Genomic DNA Buffer Set (Qiagen). Genomic DNA was processed to generate 3 kb mate pair libraries, which were sequenced with 50 bp paired-end reads on an Illumina HiSeq 2000 platform (GATC, Konstanz, Germany). This resulted in 24-60 million reads per genome, which were cured from PCR replicates and mapped to a reference sequence consisting of concatenated ICE*Hptfs3* (strain B8), ICE*Hptfs4a* (strain P12), and ICE*Hptfs4b* (strain G27) sequences, using BWA [38] with default parameters. Unmapped reads were assembled de novo using Velvet [39], and ICE elements were identified by BLAST searches ( http://blast.ncbi.nlm.nih.gov/Blast.cgi). Gaps within ICE elements were closed by Sanger sequencing.

### *Software tools for analysis of* H. pylori *genome sequences*

For comparative analysis, we evaluated all complete *H. pylori* genome sequences available in GenBank at the time of initiation of the study. We used multilocus sequence typing analysis to assign all strains to the populations and subpopulations described previously [21]. To do so, partial nucleotide sequences of the housekeeping genes *atpA*, *efp*, *mutY*, *ppa*, *trpC*, *ureI* and *yphC* were concatenated for each strain and aligned with the corresponding sequences of 345 reference strains from the MLST database ( http://pubmlst.org/helicobacter), using the Muscle algorithm within MEGA5.2 [40]. All phylogenetic trees were constructed and tested by neighbor joining with MEGA5.2, using the Kimura 2-parameter model of nucleotide substitution, and 1,000 bootstrap replications. ICE elements were identified in complete or draft genome sequences using BLAST search and visualization with the Artemis Comparison Tool [41]. A chromosomal map of strain P12 was generated using CGView [42], and WebLogo [43] was used to display sequence alignments of ICE border regions.

### Genetic analysis of hpAfrica1 strains

Genomic DNA of *H. pylori* strains was prepared using a QIAamp DNA mini kit. For MLST analysis, the housekeeping genes *atpA*, *efp*, *mutY*, *ppa*, *trpC*, *ureI* and *yphC* were partially amplified by PCR, using the primer sets described in the MLST database ( http://pubmlst.org/helicobacter), and the PCR products were sequenced. Sequences were trimmed to the required sizes, concatenated and analyzed for clustering, as described above. For examination of the right junctions of ICE*Hptfs4* islands, PCR fragments were amplified with a PANScript DNA polymerase (PAN Biotech, Aidenbach, Germany) under standard conditions in the presence of 3 mM MgCl_2_ and at an annealing temperature of 52°C, using primers WS606 (5′-AGCAATAAAACGCTTAAAAGTCTC-3′) and WS539 (5′-ATGTCCAGTAAGGAATTTGTC-3′), and subsequently analyzed by gel electrophoresis.

### GenBank accession numbers

The accession numbers for the ICE*Hptfs3* and ICE*HPtfs4* sequences determined in thuis study are as follows: 166_ICE*Hptfs4c* [GenBank:KF861855]; 175_ICE*Hptfs3* [GenBank:KF861857]; 175_ICE*Hptfs4b* [GenBank:KF861858]; 175_ICE*Hptfs4c* [GenBank:KF861859]; 328_ICE*Hptfs4a* [GenBank:KF861860]; 328_ICE*Hptfs4b* [GenBank:KF861861]; ATCC43526_ICE*Hptfs3/4a* [GenBank:KF861862]; ATCC43526_ICE*Hptfs4a* [GenBank:KF861863]; P1_ICE*Hptfs3* [GenBank:KF861854]; P1_ICE*Hptfs4b* [GenBank:KF861856]; 1_17C_ICE*Hptfs4b* [GenBank:KF861864]; 6_17A_ICE*Hptfs4b* [GenBank:KF861865]; 6_28C_ICE*Hptfs4b* [GenBank:KF861866]. Sequences of other ICE elements can be found in GenBank under the strain designations and at the genome positions shown in Table 1.

### Availability of supporting data

The phylogenetic trees shown in Figures 2 and 3 have been deposited in TreeBASE and can be accessed under http://purl.org/phylo/treebase/phylows/study/TB2:S15635.

## Competing interests

The authors declare that they have no competing interests.

## Authors’ contributions

WF conceived of and participated in the design of the study, analysed sequence data and wrote the manuscript. UB carried out the molecular genetic studies. BK and CS participated in sequence analysis. SIS participated in strain isolation and selection. RH participated in the design of the study and helped to draft the manuscript. All authors read and approved the final manuscript.

## Supplementary Material

Additional file 1: Figure S1 Gene arrangement of the plasticity zones in *H. pylori* strains 26695 and J99. Both strains contain highly rearranged truncated versions, presumably resulting from consecutive integration of 2-3 islands (ICE*Hptfs3*, ICE*Hptfs4a*, ICE*Hptfs4b*) and subsequent rearrangements, some of which are associated with insertion elements (IS605), as indicated. Click here for file

Additional file 2: Figure S2 Neighbor-joining trees of conserved type IV secretion genes. (A) Phylogenetic tree calculated from concatenated *virB9*, *virB11* and *virD4* ortholog sequences of ICE*Hptfs3* elements. (B) Phylogenetic tree calculated from concatenated *virB9*, *virB11* and *virD4* ortholog sequences of ICE*Hptfs4* elements. Click here for file

Additional file 3: Figure S3 Alignments of truncated ICE*Hptfs3* and ICE*Hptfs4* elements. (A) ICE*Hptfs3* elements are shown in comparison to ICE*Hptfs3* from strain PeCan18 (gene designation according to [15]). Additional specific genes inserted in certain elements only are shown in blue. fic, gene encoding Fic family protein similar to conserved hypothetical proteins found in *Neisseria spp*.; fic*, gene encoding Fic family protein similar to *H. pylori* chromosome-encoded proteins (e.g., JHP651). (B) Truncated ICE*Hptfs4a*, ICE*Hptfs4b* and ICE*Hptfs4c* elements are shown in comparison to the complete elements found in strains P12 and G27, respectively. Asterisks within genes indicate frameshift or nonsense mutations; blue arrows indicate truncations or rearrangements at internal AAGAATG motifs. Click here for file

Additional file 4: Figure S4 Evidence for homologous recombination between methylase/helicase orthologs in hybrid ICE*Hptfs3*/ICE*Hptfs4* elements. (A) The gene arrangement of a hybrid ICE element in strain SJM180 is compared with the ICE*Hptfs4a* element from strain P12 and the ICE*Hptfs3* element from strain PeCan18. Note that the putative DNA methylase/helicase ortholog might originate from either ICE*Hptfs3* (*pz21*) or ICE*Hptfs4a* (*hpp12_447*). Gene colouring is analogous to Figure 1 and Additional file 3: Figure S3, and frameshift or nonsense mutations are indicated by asterisks. (B) Hypothetical steps for generation of the SJM180 gene arrangement shown in (A). First, insertion of an ICE*Hptfs3* element (red) into an already integrated ICE*Hptfs4a* element (blue) generates a composite element. Subsequently, homologous recombination between the *pz21* and *hpp12*_447 orthologs and an independent truncation close to the ICE*Hptfs4a* right junction result in the deletions observed. Similar recombination events might have generated the hybrid ICE element arrangements in strains 51, Puno 135 and J99 (data not shown). Click here for file

Additional file 5: Figure S5 Border sequences of ICE*Hptfs3* (A) and ICE*Hptfs4* (B) elements. Genome positions within the respective sequences are indicated; sequences of the islands are printed in italics, and the duplicated integration motifs in bold and red. Click here for file

## References

[B1] Monack DM Mueller A Falkow S **Persistent bacterial infections: the interface of the pathogen and the host immune system** Nat Rev Microbiol 2004 2 747 765 10.1038/nrmicro955 15372085

[B2] Suerbaum S Michetti P** *Helicobacter pylori * ****infection** N Engl J Med 2002 347 1175 1186 10.1056/NEJMra020542 12374879

[B3] Peek RM Jr Blaser MJ** *Helicobacter pylori * ****and gastrointestinal tract adenocarcinomas** Nat Rev Cancer 2002 2 28 37 10.1038/nrc703 11902583

[B4] Suerbaum S Josenhans C** *Helicobacter pylori * ****evolution and phenotypic diversification in a changing host** Nat Rev Microbiol 2007 5 441 452 10.1038/nrmicro1658 17505524

[B5] Oh JD Kling-Bäckhed H Giannakis M Xu J Fulton RS Fulton LA Cordum HS Wang C Elliott G Edwards J Mardis ER Engstrand LG Gordon JI **The complete genome sequence of a chronic atrophic gastritis **** *Helicobacter pylori * ****strain: evolution during disease progression** Proc Natl Acad Sci USA 2006 103 9999 10004 10.1073/pnas.0603784103 16788065 PMC1480403

[B6] Thiberge JM Boursaux-Eude C Lehours P Dillies MA Creno S Coppée JY Rouy Z Lajus A Ma L Burucoa C Ruskoné-Foumestraux A Courillon-Mallet A De Reuse H Boneca IG Lamarque D Mégraud F Delchier JC Médigue C Bouchier C Labigne A Raymond J **From array-based hybridization of **** *Helicobacter pylori * ****isolates to the complete genome sequence of an isolate associated with MALT lymphoma** BMC Genomics 2010 11 368 10.1186/1471-2164-11-368 20537153 PMC3091627

[B7] Giannakis M Chen SL Karam SM Engstrand L Gordon JI** *Helicobacter pylori * ****evolution during progression from chronic atrophic gastritis to gastric cancer and its impact on gastric stem cells** Proc Natl Acad Sci USA 2008 105 4358 4363 10.1073/pnas.0800668105 18332421 PMC2393758

[B8] Dorer MS Fero J Salama NR **DNA damage triggers genetic exchange in **** *Helicobacter pylori* **PLoS Pathog 2010 6 e1001026 10.1371/journal.ppat.1001026 20686662 PMC2912397

[B9] Dorer MS Cohen IE Sessler TH Fero J Salama NR **Natural Competence Promotes **** *Helicobacter pylori * ****Chronic Infection** Infect Immun 2013 81 209 215 10.1128/IAI.01042-12 23115044 PMC3536137

[B10] Kraft C Stack A Josenhans C Niehus E Dietrich G Correa P Fox JG Falush D Suerbaum S **Genomic changes during chronic **** *Helicobacter pylori * ****infection** J Bacteriol 2006 188 249 254 10.1128/JB.188.1.249-254.2006 16352841 PMC1317581

[B11] Fischer W Windhager L Rohrer S Zeiller M Karnholz A Hoffmann R Zimmer R Haas R **Strain-specific genes of **** *Helicobacter pylori* ****: genome evolution driven by a novel type IV secretion system and genomic island transfer** Nucleic Acids Res 2010 38 6089 6101 10.1093/nar/gkq378 20478826 PMC2952849

[B12] Kawai M Furuta Y Yahara K Tsuru T Oshima K Handa N Takahashi N Yoshida M Azuma T Hattori M Uchiyama I Kobayashi I **Evolution in an oncogenic bacterial species with extreme genome plasticity: **** *Helicobacter pylori * ****East Asian genomes** BMC Microbiol 2011 11 104 10.1186/1471-2180-11-104 21575176 PMC3120642

[B13] Lu W Wise MJ Tay CY Windsor HM Marshall BJ Peacock C Perkins T **Comparative Analysis of the Full Genome of **** *Helicobacter pylori * ****Isolate Sahul64 Identifies Genes of High Divergence** J Bacteriol 2014 196 1073 1083 10.1128/JB.01021-13 24375107 PMC3957704

[B14] Alm RA Ling LS Moir DT King BL Brown ED Doig PC Smith DR Noonan B Guild BC deJonge BL Carmel G Tummino PJ Caruso A Uria-Nickelsen M Mills DM Ives C Gibson R Merberg D Mills SD Jiang Q Taylor DE Vovis GF Trust TJ **Genomic-sequence comparison of two unrelated isolates of the human gastric pathogen **** *Helicobacter pylori* **Nature 1999 397 176 180 10.1038/16495 9923682

[B15] Kersulyte D Velapatino B Mukhopadhyay AK Cahuayme L Bussalleu A Combe J Gilman RH Berg DE **Cluster of type IV secretion genes in **** *Helicobacter pylori* ****'s plasticity zone** J Bacteriol 2003 185 3764 3772 10.1128/JB.185.13.3764-3772.2003 12813069 PMC161572

[B16] Kersulyte D Lee W Subramaniam D Anant S Herrera P Cabrera L Balqui J Barabas O Kalia A Gilman RH Berg DE** *Helicobacter pylori* ****'s plasticity zones are novel transposable elements** PLoS ONE 2009 4 e6859 10.1371/journal.pone.0006859 19727398 PMC2731543

[B17] Alvi A Devi SM Ahmed I Hussain MA Rizwan M Lamouliatte H Mégraud F Ahmed N **Microevolution of **** *Helicobacter pylori * ****type IV secretion systems in an ulcer disease patient over a ten-year period** J Clin Microbiol 2007 45 4039 4043 10.1128/JCM.01631-07 17942650 PMC2168557

[B18] Lu H Hsu PI Graham DY Yamaoka Y **Duodenal ulcer promoting gene of **** *Helicobacter pylori* **Gastroenterology 2005 128 833 848 10.1053/j.gastro.2005.01.009 15825067 PMC3130061

[B19] Jung SW Sugimoto M Shiota S Graham DY Yamaoka Y **The intact **** *dupA * ****cluster is a more reliable **** *Helicobacter pylori * ****virulence marker than **** *dupA * ****alone** Infect Immun 2012 80 381 387 10.1128/IAI.05472-11 22038914 PMC3255691

[B20] Lehours P Dupouy S Bergey B Ruskoné-Foumestraux A Delchier JC Rad R Richy F Tankovic J Zerbib F Mégraud F Ménard A **Identification of a genetic marker of **** *Helicobacter pylori * ****strains involved in gastric extranodal marginal zone B cell lymphoma of the MALT-type** Gut 2004 53 931 937 10.1136/gut.2003.028811 15194637 PMC1774103

[B21] Falush D Wirth T Linz B Pritchard JK Stephens M Kidd M Blaser MJ Graham DY Vacher S Perez-Perez GI Yamaoka Y Mégraud F Otto K Reichard U Katzowitsch E Wang X Achtman M Suerbaum S **Traces of human migrations in **** *Helicobacter pylori * ****populations** Science 2003 299 1582 1585 10.1126/science.1080857 12624269

[B22] Linz B Balloux F Moodley Y Manica A Liu H Roumagnac P Falush D Stamer C Prugnolle F van der Merwe SW Yamaoka Y Graham DY Perez-Trallero E Wadström T Suerbaum S Achtman M **An African origin for the intimate association between humans and **** *Helicobacter pylori* **Nature 2007 445 915 918 10.1038/nature05562 17287725 PMC1847463

[B23] Moodley Y Linz B Yamaoka Y Windsor HM Breurec S Wu JY Maady A Bernhöft S Thiberge JM Phuanukoonnon S Jobb G Siba P Graham DY Marshall BJ Achtman M **The peopling of the Pacific from a bacterial perspective** Science 2009 323 527 530 10.1126/science.1166083 19164753 PMC2827536

[B24] Gressmann H Linz B Ghai R Pleissner KP Schlapbach R Yamaoka Y Kraft C Suerbaum S Meyer TF Achtman M **Gain and loss of multiple genes during the evolution of **** *Helicobacter pylori* **PLoS Genet 2005 1 e43 10.1371/journal.pgen.0010043 16217547 PMC1245399

[B25] Furuta Y Kawai M Yahara K Takahashi N Handa N Tsuru T Oshima K Yoshida M Azuma T Hattori M Uchiyama I Kobayashi I **Birth and death of genes linked to chromosomal inversion** Proc Natl Acad Sci USA 2011 108 1501 1506 10.1073/pnas.1012579108 21212362 PMC3029772

[B26] Duncan SS Valk PL McClain MS Shaffer CL Metcalf JA Bordenstein SR Cover TL **Comparative genomic analysis of East Asian and non-Asian **** *Helicobacter pylori * ****strains identifies rapidly evolving genes** PLoS ONE 2013 8 e55120 10.1371/journal.pone.0055120 23383074 PMC3561388

[B27] Duncan SS Bertoli MT Kersulyte D Valk PL Tamma S Segal I McClain MS Cover TL Berg DE **Genome Sequences of Three hpAfrica2 Strains of **** *Helicobacter pylori* **Genome Announc 2013 1 e00729 13 24072860 10.1128/genomeA.00729-13 PMC3784780

[B28] Kersulyte D Rossi M Berg DE **Sequence Divergence and Conservation in Genomes of **** *Helicobacter cetorum * ****Strains from a Dolphin and a Whale** PLoS One 2013 8 e83177 10.1371/journal.pone.0083177 24358262 PMC3866246

[B29] Vermoote M Vandekerckhove TT Flahou B Pasmans F Smet A De Groote D Van Criekinge W Ducatelle R Haesebrouck F **Genome sequence of **** *Helicobacter suis * ****supports its role in gastric pathology** Vet Res 2011 42 51 10.1186/1297-9716-42-51 21414191 PMC3065412

[B30] Olbermann P Josenhans C Moodley Y Uhr M Stamer C Vauterin M Suerbaum S Achtman M Linz B **A global overview of the genetic and functional diversity in the **** *Helicobacter pylori cag * ****pathogenicity island** PLoS Genet 2010 6 e1001069 10.1371/journal.pgen.1001069 20808891 PMC2924317

[B31] Wozniak RA Waldor MK **Integrative and conjugative elements: mosaic mobile genetic elements enabling dynamic lateral gene flow** Nat Rev Microbiol 2010 8 552 563 10.1038/nrmicro2382 20601965

[B32] Grove JI Alandiyjany MN Delahay RM **Site-specific Relaxase Activity of a VirD2-like Protein Encoded within the **** *tfs4 * ****Genomic Island of **** *Helicobacter pylori* **J Biol Chem 2013 288 26385 26396 10.1074/jbc.M113.496430 23900838 PMC3772185

[B33] Menard KL Grossman AD **Selective pressures to maintain attachment site specificity of integrative and conjugative elements** PLoS Genet 2013 9 e1003623 10.1371/journal.pgen.1003623 23874222 PMC3715440

[B34] Roberts AP Mullany P **A modular master on the move: the Tn**** *916 * ****family of mobile genetic elements** Trends Microbiol 2009 17 251 258 10.1016/j.tim.2009.03.002 19464182

[B35] Mullany P Williams R Langridge GC Turner DJ Whalan R Clayton C Lawley T Hussain H McCurrie K Morden N Allan E Roberts AP **Behavior and target site selection of conjugative transposon Tn916 in two different strains of toxigenic **** *Clostridium difficile* **Appl Environ Microbiol 2012 78 2147 2153 10.1128/AEM.06193-11 22267673 PMC3302608

[B36] Kim DJ Park KS Kim JH Yang SH Yoon JY Han BG Kim HS Lee SJ Jang JY Kim KH Kim MJ Song JS Kim HJ Park CM Lee SK Lee BI Suh SW** *Helicobacter pylori * ****proinflammatory protein up-regulates NF-κB as a cell-translocating Ser/Thr kinase** Proc Natl Acad Sci USA 2010 107 21418 21423 10.1073/pnas.1010153107 21098302 PMC3003084

[B37] Hussein NR Argent RH Marx CK Patel SR Robinson K Atherton JC** *Helicobacter pylori dupA * ****is polymorphic, and its active form induces proinflammatory cytokine secretion by mononuclear cells** J Inf Dis 2010 202 261 269 10.1086/653587 20533870

[B38] Li H Durbin R **Fast and accurate short read alignment with Burrows-Wheeler transform** Bioinformatics 2009 25 1754 1760 10.1093/bioinformatics/btp324 19451168 PMC2705234

[B39] Zerbino DR Birney E **Velvet: algorithms for de novo short read assembly using de Bruijn graphs** Genome Res 2008 18 821 829 10.1101/gr.074492.107 18349386 PMC2336801

[B40] Tamura K Dudley J Nei M Kumar S **MEGA4: Molecular Evolutionary Genetics Analysis (MEGA) software version 4.0** Mol Biol Evol 2007 24 1596 1599 10.1093/molbev/msm092 17488738

[B41] Carver TJ Rutherford KM Berriman M Rajandream MA Barrell BG Parkhill J **ACT: the Artemis Comparison Tool** Bioinformatics 2005 21 3422 3423 10.1093/bioinformatics/bti553 15976072

[B42] Stothard P Wishart DS **Circular genome visualization and exploration using CGView** Bioinformatics 2005 21 537 539 10.1093/bioinformatics/bti054 15479716

[B43] Crooks GE Hon G Chandonia JM Brenner SE **WebLogo: a sequence logo generator** Genome Res 2004 14 1188 1190 10.1101/gr.849004 15173120 PMC419797

