# Supplementary figures and images for: A comprehensive analysis of Helicobacter pylori plasticity zones reveals that they are integrating conjugative elements with intermediate integration specificity
Source: BMC Genomics. 2014 Apr 27;15:310. doi: 10.1186/1471-2164-15-310 (PMC4234485; doi:10.1186/1471-2164-15-310)

## Slide 1
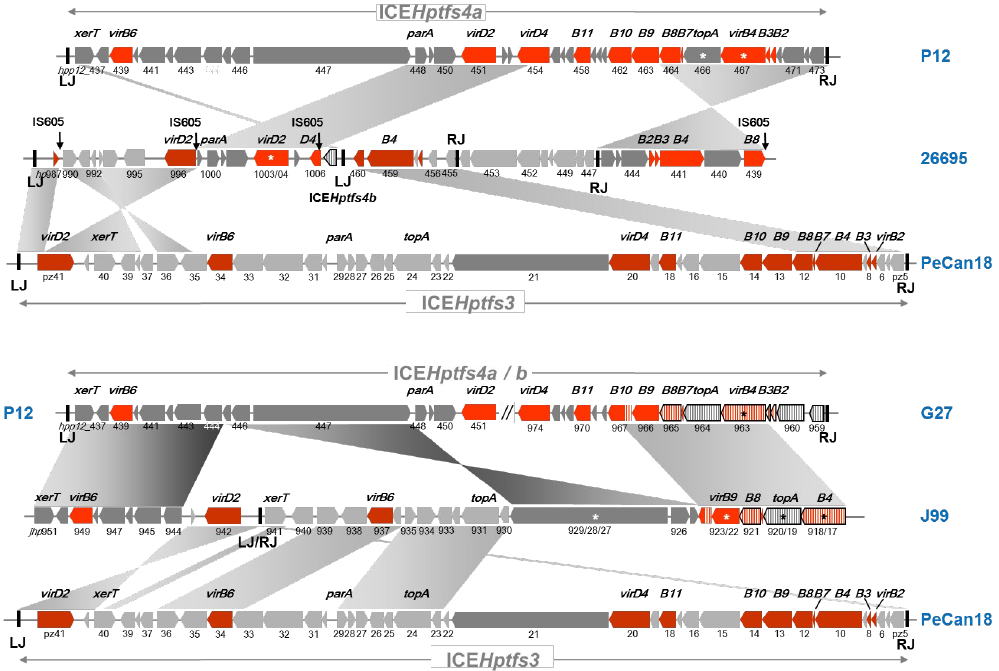

Supplement: Additional file 1: Figure S1 — Gene arrangement of the plasticity zones in H. pylori strains 26695 and J99. Both strains contain highly rearranged truncated versions, presumably resulting from consecutive integration of 2-3 islands (ICEHptfs3, ICEHptfs4a, ICEHptfs4b) and subsequent rearrangements, some of which are associated with insertion elements (IS605), as indicated. [file 1471-2164-15-310-S1.pptx]

## Slide 1
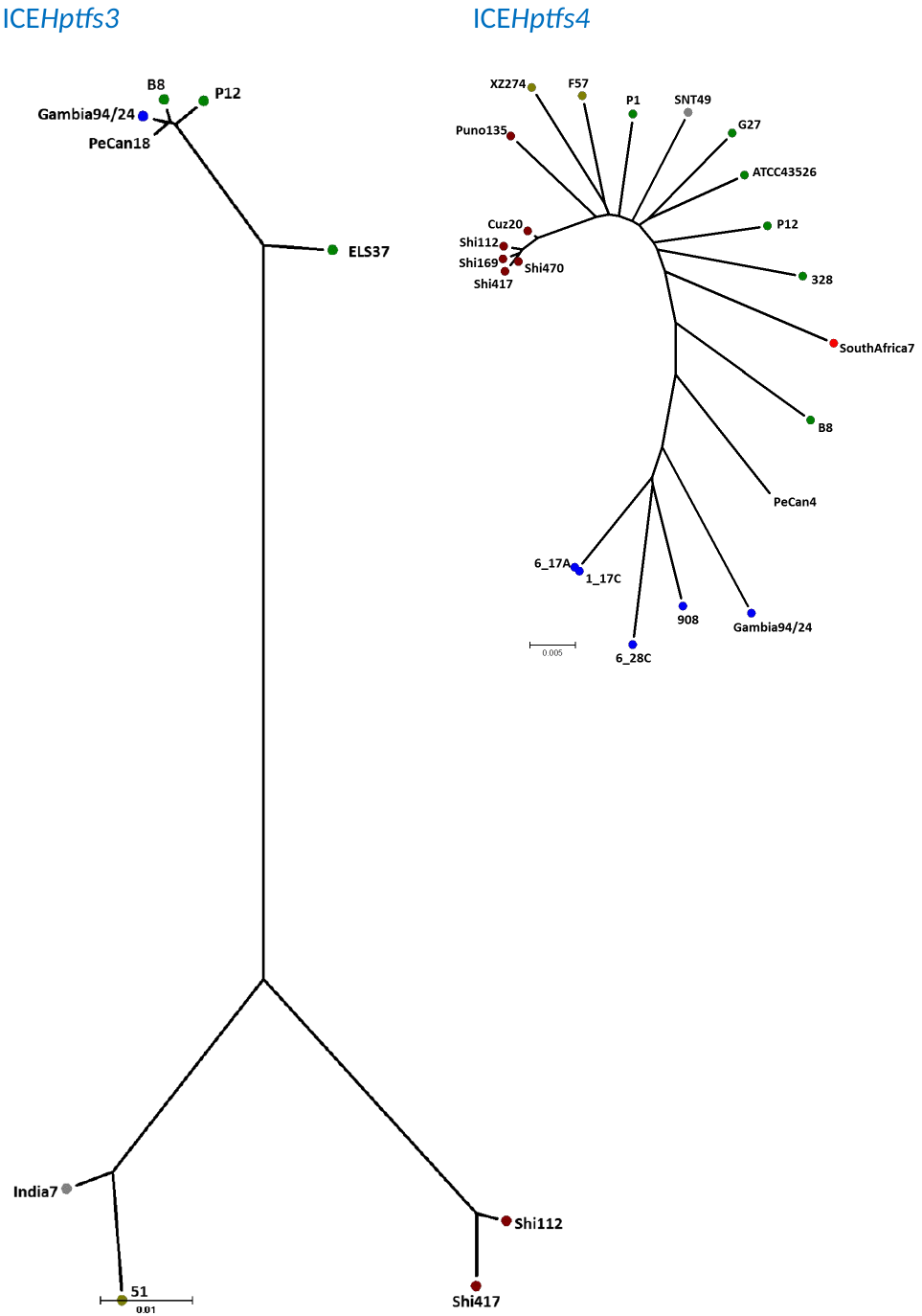

ICEHptfs3
ICEHptfs4

Supplement: Additional file 2: Figure S2 — Neighbor-joining trees of conserved type IV secretion genes. (A) Phylogenetic tree calculated from concatenated virB9, virB11 and virD4 ortholog sequences of ICEHptfs3 elements. (B) Phylogenetic tree calculated from concatenated virB9, virB11 and virD4 ortholog sequences of ICEHptfs4 elements. [file 1471-2164-15-310-S2.pptx]

## Slide 1
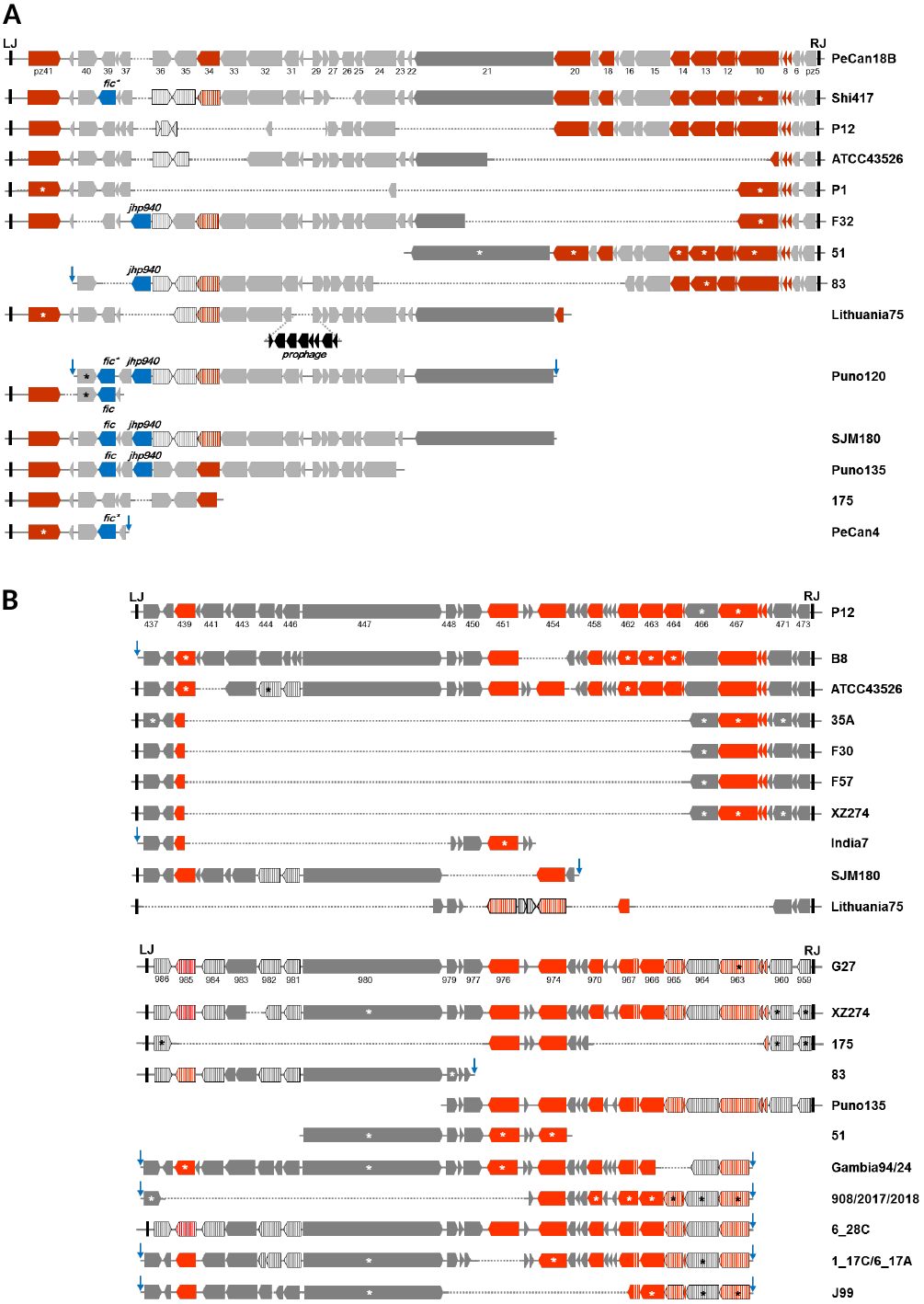

A
B

Supplement: Additional file 3: Figure S3 — Alignments of truncated ICEHptfs3 and ICEHptfs4 elements. (A) ICEHptfs3 elements are shown in comparison to ICEHptfs3 from strain PeCan18 (gene designation according to [15]). Additional specific genes inserted in certain elements only are shown in blue. fic, gene encoding Fic family protein similar to conserved hypothetical proteins found in Neisseria spp.; fic*, gene encoding Fic family protein similar to H. pylori chromosome-encoded proteins (e.g., JHP651). (B) Truncated ICEHptfs4a, ICEHptfs4b and ICEHptfs4c elements are shown in comparison to the complete elements found in strains P12 and G27, respectively. Asterisks within genes indicate frameshift or nonsense mutations; blue arrows indicate truncations or rearrangements at internal AAGAATG motifs. [file 1471-2164-15-310-S3.pptx]

## Slide 1
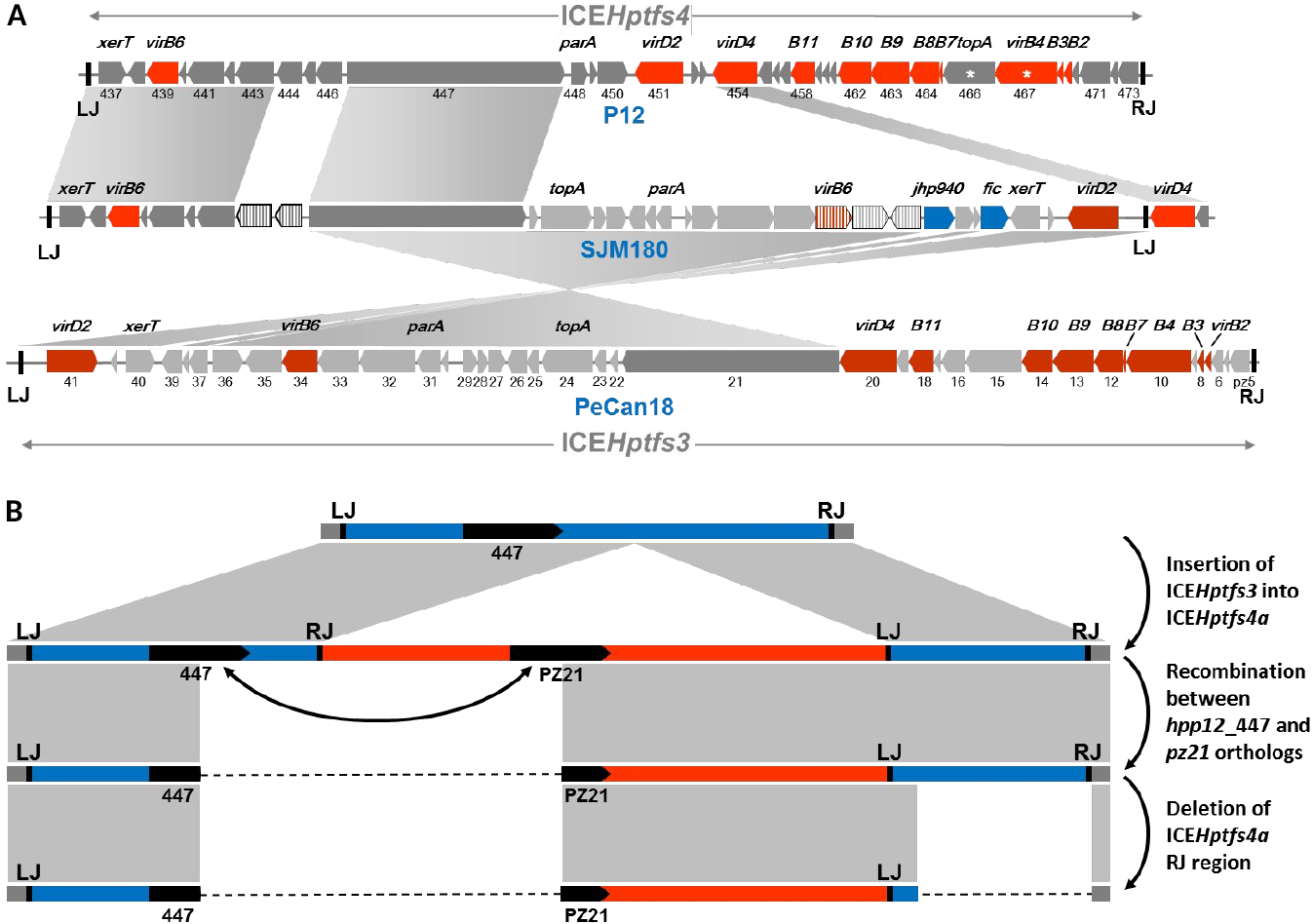

A
B

Supplement: Additional file 4: Figure S4 — Evidence for homologous recombination between methylase/helicase orthologs in hybrid ICEHptfs3/ICEHptfs4 elements. (A) The gene arrangement of a hybrid ICE element in strain SJM180 is compared with the ICEHptfs4a element from strain P12 and the ICEHptfs3 element from strain PeCan18. Note that the putative DNA methylase/helicase ortholog might originate from either ICEHptfs3 (pz21) or ICEHptfs4a (hpp12_447). Gene colouring is analogous to Figure 1 and Additional file 3: Figure S3, and frameshift or nonsense mutations are indicated by asterisks. (B) Hypothetical steps for generation of the SJM180 gene arrangement shown in (A). First, insertion of an ICEHptfs3 element (red) into an already integrated ICEHptfs4a element (blue) generates a composite element. Subsequently, homologous recombination between the pz21 and hpp12_447 orthologs and an independent truncation close to the ICEHptfs4a right junction result in the deletions observed. Similar recombination events might have generated the hybrid ICE element arrangements in strains 51, Puno 135 and J99 (data not shown). [file 1471-2164-15-310-S4.pptx]

## Slide 1
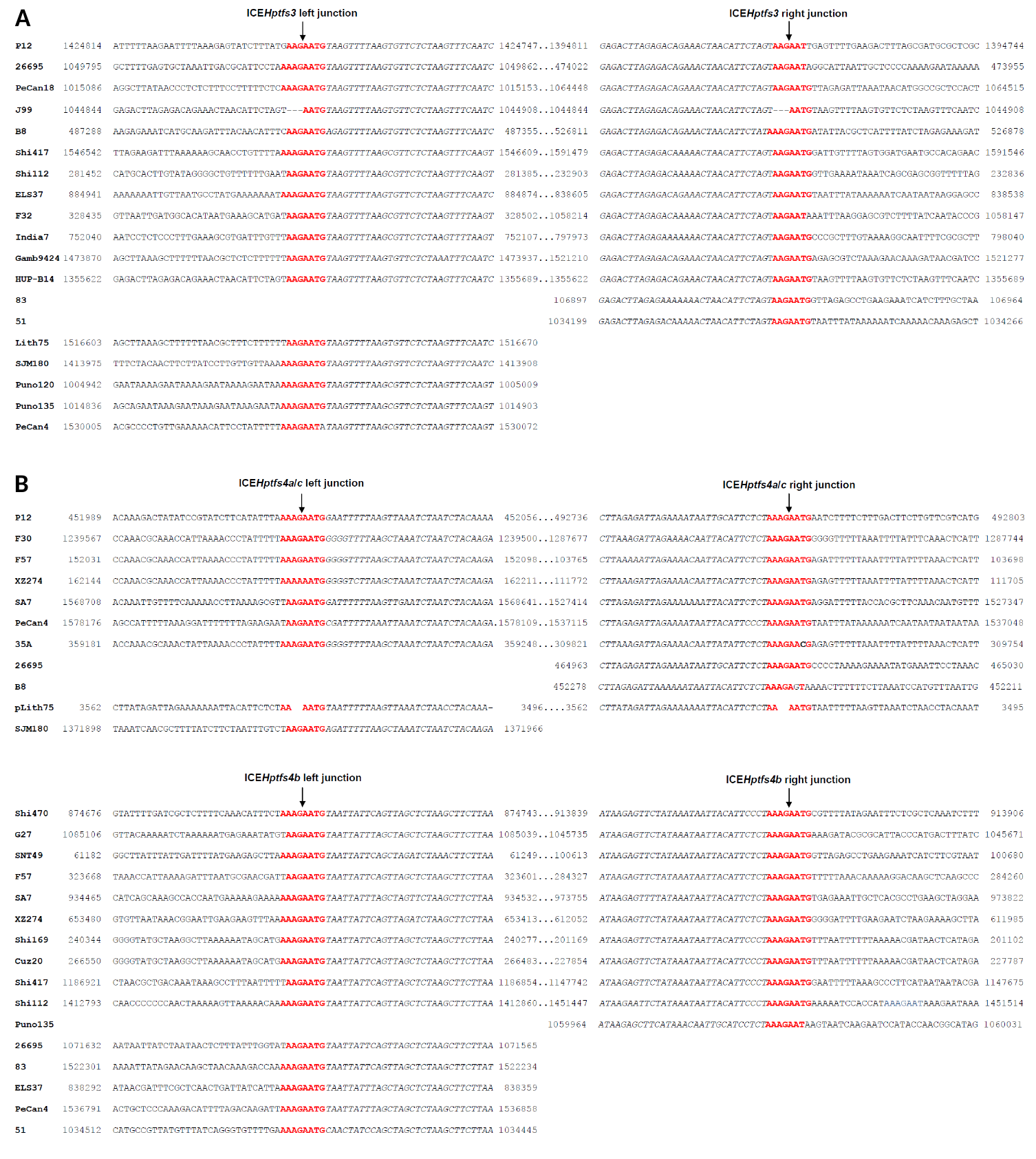

A
B

Supplement: Additional file 5: Figure S5 — Border sequences of ICEHptfs3 (A) and ICEHptfs4 (B) elements. Genome positions within the respective sequences are indicated; sequences of the islands are printed in italics, and the duplicated integration motifs in bold and red. [file 1471-2164-15-310-S5.pptx]
